# Electrochemical and Solution Structural Characterization of Fe(III) Azotochelin Complexes: Examining the Coordination Behavior of a Tetradentate Siderophore

**DOI:** 10.1021/acs.inorgchem.2c02777

**Published:** 2022-10-17

**Authors:** Natalia G. Baranska, Alison Parkin, Anne-K. Duhme-Klair

**Affiliations:** Department of Chemistry, University of York, Heslington, York YO10 5DD, United Kingdom

## Abstract

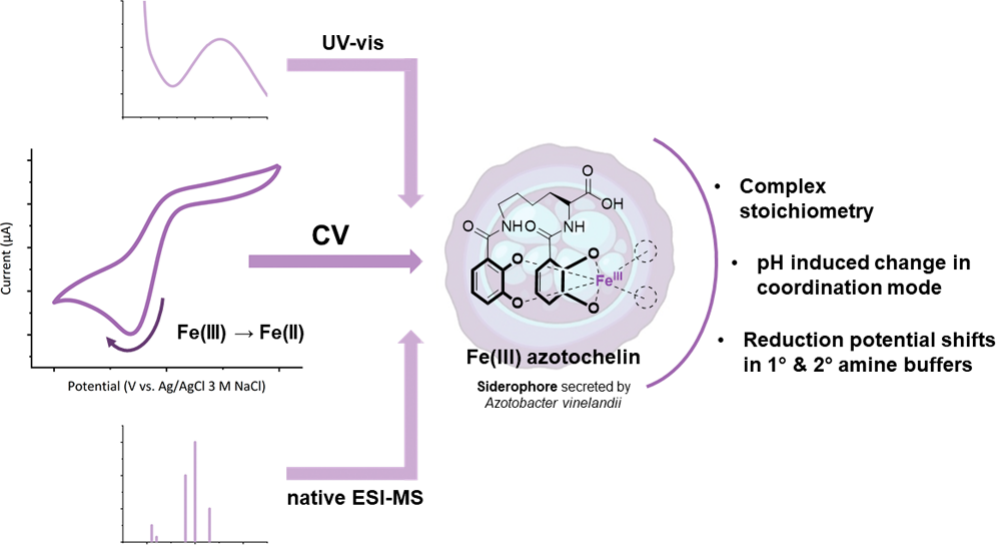

We report an electrochemical setup comprising a boron-doped diamond (BDD) working electrode for the electrochemical study of iron(III) catecholate siderophores. We demonstrate its successful application in the voltammetric investigation of iron(III) azotochelin, an iron complex of a bis(catecholate) siderophore. Cyclic voltammetry results, when complemented by UV–vis and native electrospray ionization-mass spectrometry (ESI-MS) characterization, reveal the formation of a coordinatively unsaturated tetracoordinate 1:1 complex of Fe:azotochelin (M_1_:L_1_) at neutral pH, contrary to iron(III) tetradentate siderophore complexes of other classes which favor the hexacoordinate environment of an M_2_:L_3_ species. A notable effect of pH and buffer composition on the reduction potential of iron(III) azotochelin is demonstrated. Lower pH values and buffers encompassing primary or secondary amines facilitate a positive potential shift of up to +290 mV and +250 mV vs Ag/AgCl 3 M NaCl, respectively. The study was extended to the investigation of the iron(III) complexes of hexadentate siderophores. For tris(catecholate) siderophores, enterobactin and protochelin, the reduction potentials were found to lie beyond the potential window accessible to the BDD electrode; however, we were successful in observing the electrochemical behavior of a tris(hydroxamate) siderophore, ferricrocin.

## Abstract 2

The iron(III) complex of azotochelin, a tetradentate bis(catecholate) siderophore, was investigated by voltammetry at a boron-doped diamond electrode, UV−vis spectroscopy, and native ESI-mass spectrometry. The data reveals formation of a coordinatively unsaturated tetracoordinate M_1_:L_1_ complex at pH 7.0. Significant positive reduction potential shifts are observed at pH < 6.25 and in buffers containing primary and secondary amines, respectively. The behavior at a lower pH is consistent with intramolecular changes in the coordination mode rather than complex stoichiometry.

## Introduction

Iron is an essential element within biological systems, a role which is attributed to its extensive and flexible coordination capabilities which confer upon it an ability to exist in a number of different coordination states under physiological conditions.^1^ Despite its importance in biology, iron(III) possesses extremely low solubility in aqueous aerobic environments at pH 7.0 (*K*_sp_ [Fe^3+^][OH^–^]^3^ = 10^–38.7^ M), leading to its limited bioavailability.^2^ To overcome this, microorganisms employ siderophores, low molecular weight chelators, to facilitate the solubilization of iron(III) from the environment and its transport across the cellular membrane.^3^ Siderophores incorporate ligands with hard donor oxygen atoms to selectively coordinate the highly Lewis acidic iron(III), yielding complexes of extremely high thermodynamic stability (log *K*_f_ ≅ 30–49).^4^ However, iron in such a strongly chelated form cannot be utilized by the cell, and therefore, microorganisms have evolved efficient mechanisms to disassemble these metal-siderophore complexes and allow the intracellular release of iron. The crucial role of siderophores in bacterial iron transport, along with their ability to form strong complexes with their cognate periplasmic binding proteins, has prompted their applications in the fields of medicinal chemistry and artificial metalloenzymes.^5−8^

Redox-initiated metal release has been the most widely hypothesized and investigated mechanism for the in vivo intracellular release of iron from its siderophore complexes.^9−12^ Upon reduction of the iron(III) metal center, its charge density decreases, while the ionic radius increases, lessening its Lewis acid character and subsequently resulting in a decreased affinity of oxygen donor atoms toward it.^12^ This results in iron(II) siderophore complexes that exhibit much lower thermodynamic stability compared to their iron(III) equivalents.^12^ Consequently, the iron(II) siderophores are more susceptible to ligand exchange and complex dissociation, facilitating iron release. However, the high stability of iron(III) siderophore complexes leads to very negative redox potentials being associated with the Fe(III)/(II) redox couple (−350 mV to −750 mV vs normal hydrogen electrode (NHE) at pH 7.0), placing them beyond the range accessible to most biological reducing agents under physiological conditions, such as nicotinamide adenine dinucleotide phosphate (NADPH) (−324 mV vs NHE at pH 7.0) and nicotinamide adenine dinucleotide (NADH) (−320 mV vs NHE at pH 7.0).^4,13^ Numerous electrochemical studies have been performed to identify factors capable of modulating the reduction potentials of these complexes and hence probing the feasibility of the reductive metal siderophore release mechanism.

**Figure 1:**
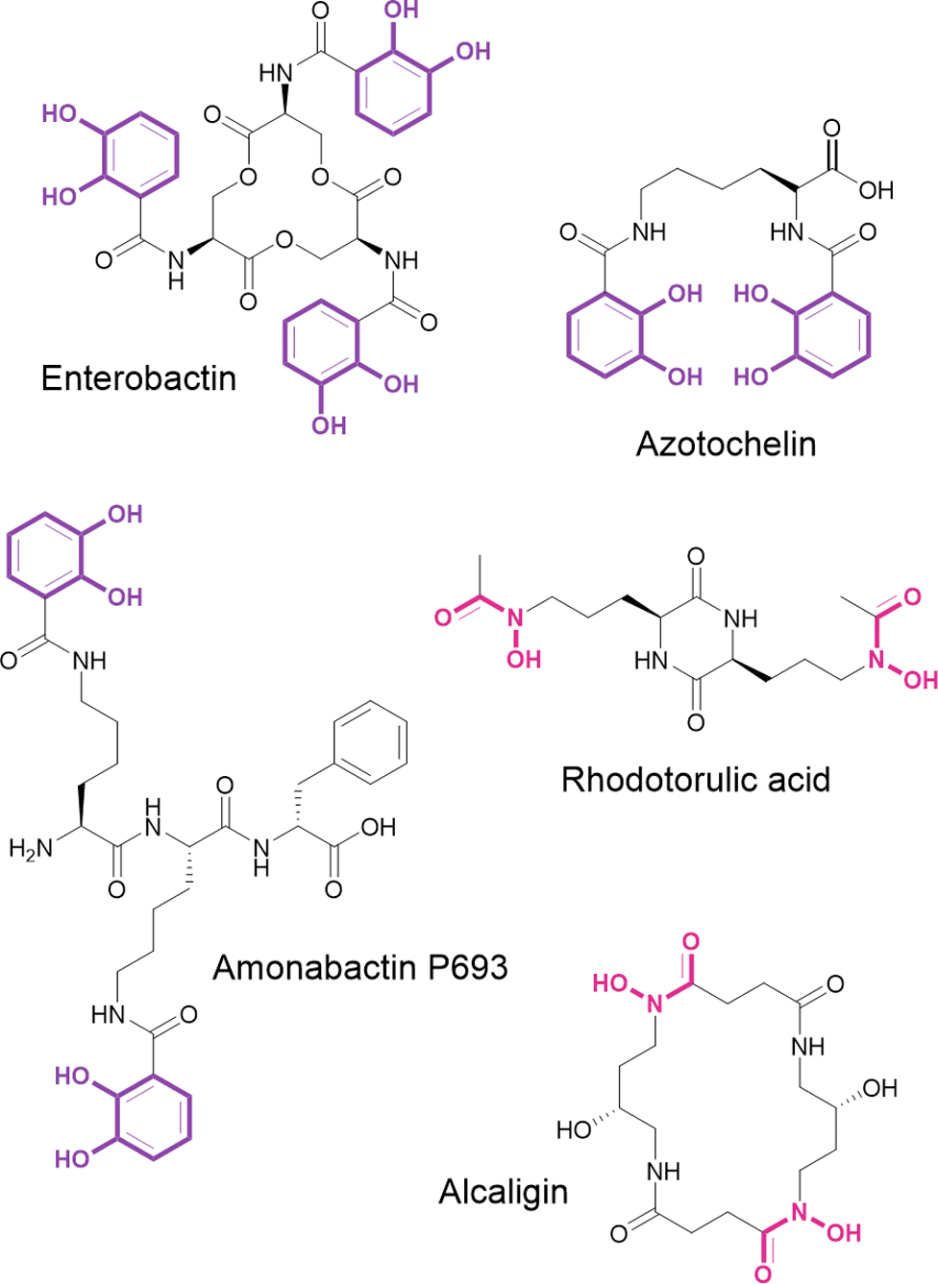
Chemical structures of siderophores discussed within the text, with their respective iron binding units highlighted in color. Catechol groups are highlighted in purple, and hydroxamate groups are highlighted in pink.

Pioneering contributions to the field of catecholate (cat) siderophore electrochemistry have been made by Raymond and co-workers, with an emphasis on the redox properties of the hexadentate siderophore, enterobactin (Figure 1), and its synthetic analogues.^9,10,14−17^ These studies illustrated the feasibility of using mercury drop electrodes (MDEs) to measure the Fe(III)/(II) reduction potential of iron(III) siderophore complexes and since then MDEs have become the most commonly employed working electrode across published investigations of these chelators.^9,18−21^

**Figure 2:**
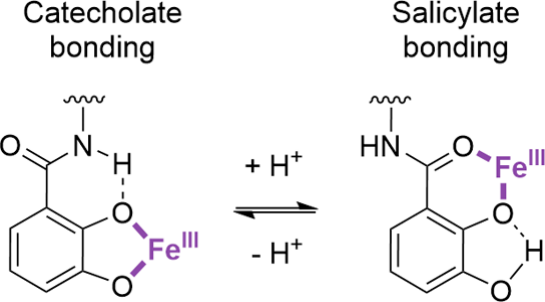
Schematic representation of the switch in coordination modes as exhibited by iron(III) catecholate siderophore complexes in response to a change in pH through the protonation of the phenolic oxygen in the meta position.

Enterobactin has attracted much attention for being a siderophore exhibiting the highest affinity toward iron(III), as indicated by its pFe value of 35.6,^14^ which defines the free iron concentration, −log [Fe^3+^], at pH 7.4 in a solution where the total [Fe^3+^] = 10^–6^ M and total [L] = 10^–5^ M. This siderophore comprises a cyclic trilactone backbone and three catecholate binding moieties (Figure 1), the strongest siderophore donor groups. As determined by solution cyclic voltammetry (CV), iron(III) enterobactin complexes exhibit a formal midpoint potential (*E*_1/2_) associated with the electrochemically reversible, one-electron Fe(III)/(II) redox couple (calculated as the average of the cathodic (*E*_pc_) and anodic (*E*_pa_) peak positions) of −790 mV vs NHE at pH 7.4.^10^ This negative potential is consistent with the high pFe value of the iron(III) enterobactin catecholate complex. A significant impact of pH on the reduction potential of iron(III) enterobactin has been documented, with a shift to more positive values being observed in response to an increasing proton concentration over the pH range 11.4 to 6.0.^10^ Under sufficiently acidic conditions, which facilitate the protonation of the meta catecholate oxygens of the iron(III) enterobactin complex (p*K*_a_ 4.95, 3.52, and 2.5), a shift to salicylate-type metal bonding has been shown to occur (Figure 2), resulting in a metal complex of lower stability and hence a more positive Fe(III)/(II) reduction potential, a phenomenon not observed in other classes of siderophores.^17,22^ However, the pH values required to protonate the iron(III) enterobactin complex limit this iron release mechanism to acidic compartments within the bacterial cell, such as the periplasm. Presently, only one example of periplasmic release has been identified for siderophore-mediated transport, involving an iron(III)-pyoverdine complex (*Pseudomonas aeruginosa*); conversely, numerous studies have demonstrated the occurrence of the iron reductive mechanism within the cytoplasm, suggesting the presence of supplementary redox-mediated mechanism(s).^23−25^

**Figure 3:**
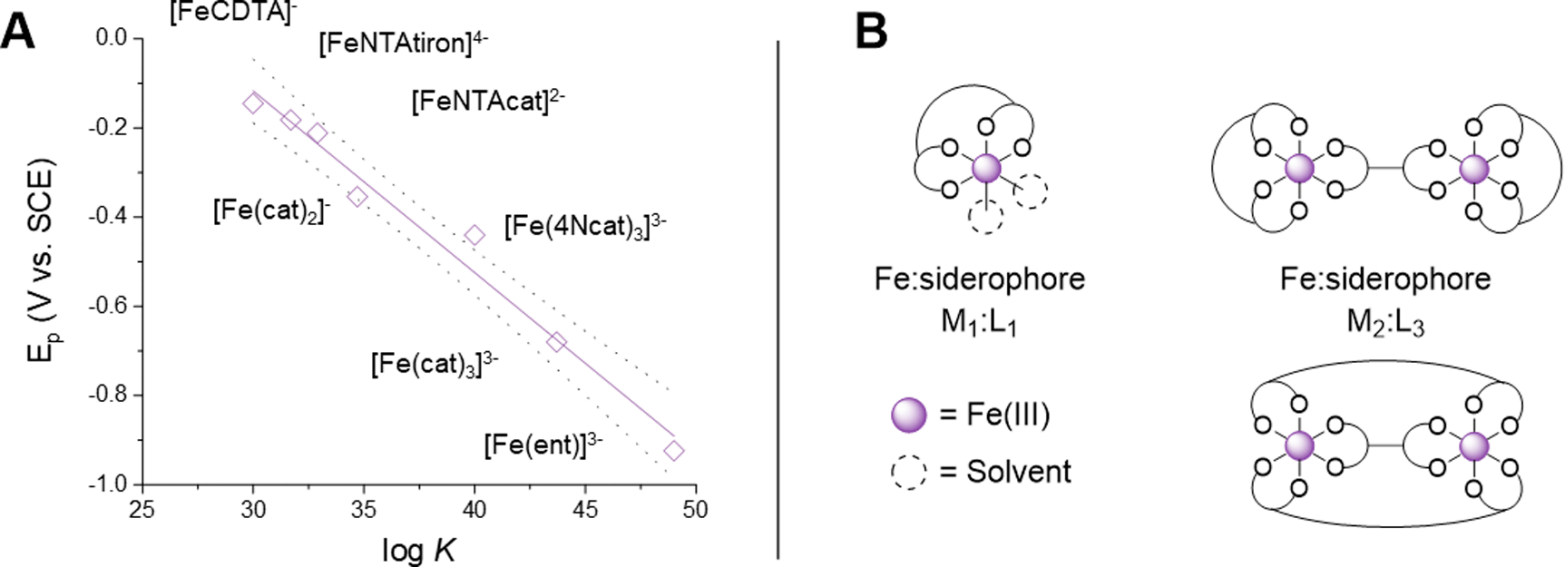
(**A**) A graphical illustration of the “chelate scale” demonstrating the relationship between the Fe^3+/2+^ reduction potential (*E*_p_) and the stability (log *K*) of iron(III) complexes with catechol ligands; CDTA = *cis*-1,2-cyclohexylenedinitrilotetraacetate, NTA = nitrilotriacetate, tiron = 4,5-dihydroxy-1,3-benzenedisulfonic acid, cat = catechol, 4Ncat = 4-nitrocatechol, ent = enterobactin. The purple solid line and the dotted lines represent the linear regression and the 95% confidence interval, respectively. Adapted from ref (27). Copyright 1994 American Chemical Society. **(B**) Model representations of the possible stoichiometries for iron(III) complexes of tetradentate siderophores.

Electrochemical studies by Crumbliss and colleagues investigated the effect of ligand denticity on the reduction potential of iron(III) siderophores.^11,26^ Experiments on iron(III) hydroxamate siderophore complexes have revealed a negative linear relationship between the denticity and iron(III) reduction potential, interpreted as a direct result of differences in thermodynamic stability among bidentate, tetradentate, and hexadentate ligands. Comparable investigations of catecholate binding groups have been limited to simple catechol (cat) ligands studied in varying metal to ligand (M:L) ratios, as a simulation of iron(III) catecholate siderophore complexes with ligands of varying denticities.^27^ Taylor et al. have used these results to develop a linear “chelate scale” (Figure 3A) that enables the estimation of stability constants for iron(III) catecholate complexes based on the metal reduction potential.

To date, natural tetradentate catecholate siderophores such as azotochelin, amonabactins (Figure 1), and salmochelins have been omitted from voltammetric studies. However, several studies have previously shown that the solution chemistries of their iron(III) complexes differ in comparison to iron(III) hydroxamates, and therefore, it can be hypothesized that their electrochemical properties may also exhibit contrasting behavior.^28,29^ Speciation studies through electrospray ionization-mass spectrometry (ESI-MS) characterization of alcaligin and rhodotorulic acid (Figure 1), cyclic and linear dihydroxamate siderophores, respectively, have shown the favorable formation of M_2_:L_3_ iron(III) complexes (Figure 3B) at neutral pH for both siderophores, despite the preparation of the stoichiometric M_1_:L_1_ solutions.^28^ The tetracoordinated M_1_:L_1_ dihydroxamate species (Figure 3B) have only been observed upon an increase in H^+^ concentration, justifying the difference in formal midpoint potentials between the iron(III) complexes of tetradentate and hexadentate hydroxamate siderophores being within 75 mV at pH 7.0, as both form hexacoordinate iron(III) species under those conditions.^26^ Conversely, structural studies of bis(catecholate) amonabactins (Figure 1) have shown the M_1_:L_1_ complex to be the predominant species at neutral pH.^29^ However, no electrochemical data has been reported for iron(III) amonabactins, nor have such investigations been documented for other iron(III) bis(catecholate) siderophores. Such studies, however, would provide an important insight into the role of these ligands in siderophore-mediated iron transport, particularly as they exhibit inferior iron chelating abilities in comparison to hexadentate siderophores, yet are still secreted by many microorganisms.^30^

The preference of (bis)catecholate siderophores to form tetracoordinate iron complexes leaves the iron coordination sphere unsaturated, with two free sites available for binding to other molecules. In an aqueous solution, these vacant sites are often occupied by aqua/hydroxo ligands. The coordination of alternative ligands at these “vacant” sites can be hypothesized to alter the chemistry of the iron(III) center, including its reduction potential, particularly if softer donor groups are utilized that stabilize iron(II) over iron(III). This has led to the speculation of ternary complex formation being a feasible pathway for the modulation of the iron(III) reduction potential in iron(III) siderophore complexes, enabling the reductive iron release mechanism.^12,31,32^ Furthermore, there have also been reports on the effects of buffers on the electrochemical behavior of iron(III) siderophores, implying their influence within the second coordination sphere; however, no detailed studies have been reported.^21,26^

Azotochelin (Az) is a bis(catecholate) siderophore (Figure 1), one of the three catecholate siderophores secreted by the nitrogen-fixing soil bacterium *Azotobacter vinelandii*.^33^ It has attracted the attention of researchers due to its ability to sequester both iron and molybdenum from the environment, predominantly prompting experiments on the chemistry of its molybdenum complex.^34,35^ The characterization of azotochelin iron(III) complexes has so far been limited to UV–vis spectroscopy with no consensus being reached on its speciation, demonstrating the need for further investigation.^35,36^ This, along with azotochelin’s ease of synthesis, its simple lysine backbone, and the absence of additional chelating groups, has prompted the selection of this siderophore for our study as a representative model for tetradentate catecholate siderophores.^37^

In this manuscript, we examine the redox properties of iron(III) azotochelin, and to the best of our knowledge, we report the electrochemical behavior of an iron(III) bis(catecholate) siderophore complex for the first time, as well as evaluate the effect of buffer compositions and pH levels on the reduction potential of iron(III) azotochelin. We employ an electrochemical setup comprising a boron-doped diamond (BDD) working electrode. A BDD working electrode has been previously implemented in the study of iron(III) phytosiderophores, based on the aminocarboxylate motif, demonstrating the ability of these electrodes to interrogate the Fe(III)/(II) redox chemistry of a siderophore complex.^38^ The solution cyclic voltammetry measurements are supplemented with native ESI-MS and UV–vis data to confirm the speciation of the complexes under investigation, highlighting the advantage of the complementary utilization of these techniques. We also perform electrochemical investigations into several hexadentate siderophores to examine the feasibility of the BDD working electrode to study iron(III) siderophore complexes of varied architecture and speciation patterns.

## Results and Discussion

### Electrochemistry of Fe(III) Azotochelin

**Figure 4:**
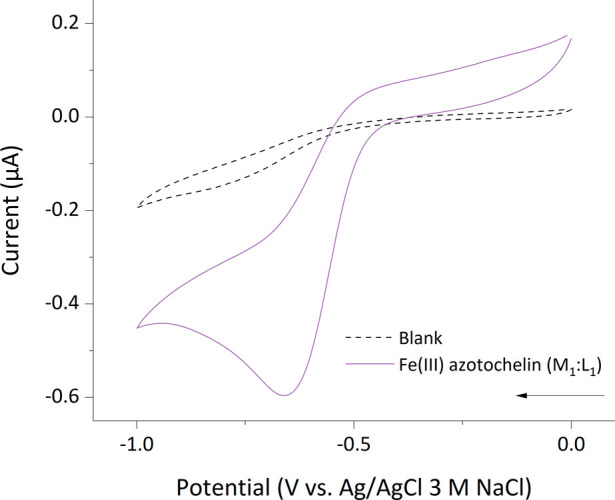
Cyclic voltammogram of an equimolar (M_1_:L_1_) iron(III) azotochelin solution in a 5 mM BIS-TRIS buffer containing 100 mM NaCl at pH 7.0. Analyte concentrations of [Fe] = 0.45 mM and [Az] = 0.45 mM; *v* = 10 mV s^–1^, *E*_step_ = 0.01 V. The arrow indicates the direction of the current.

The electrochemical behavior of a 0.45 mM iron(III) bis(catecholate) siderophore solution was examined by solution CV in a 5 mM BIS-TRIS buffer (pH 7.0), containing 100 mM NaCl as the supporting electrolyte. The voltammogram shown in Figure 4 was recorded after the sequential addition of FeCl_3_ and azotochelin stocks to a buffer solution (Figure S1), followed by an equilibration period of 5 min after each step to enable the formation of the complex in situ within the electrochemical cell. Successful complexation was concluded from the deep-purple coloration of the solution, characteristic of an iron(III) bis(catecholate) siderophore complex. The voltammogram exhibits an irreversible reduction current with a potential peak of −660 mV vs Ag/AgCl 3 M NaCl (−470 mV vs NHE), which was only apparent after the addition of azotochelin to the FeCl_3_ solution (in an M_1_:L_1_ ratio) and was therefore attributed to the Fe(III)/(II) reduction of the metal-siderophore complex. Upon increasing the concentration of iron and azotochelin, the shape of the voltammogram remained essentially unchanged; a negative shift of 10 mV was observed in the presence of excess ligand, up to a final M_1_:L_3_ ratio, with a further negative shift of 10 mV upon an increase in iron concentration which yielded a final solution of M_2_:L_3_ (Figure S2 and Table S1). The small magnitude of the reduction potential shifts leads us to conclude that the metal-siderophore complex responsible for the observed electrochemical response exhibits high stability under experimental conditions and does not undergo significant speciation changes. To investigate whether this electrochemical behavior is characteristic for bis(catecholate) siderophores, we have examined the iron(III) complex of bis(2,3-dihydroxybenzoyl-*L*-serine) (bisDHBS), an enterobactin hydrolysis product that structurally resembles azotochelin (Figure S3A). The voltammogram (Figure S3B) was obtained in conditions analogous to those used for the study of azotochelin, employing an equimolar ratio of M:L. We have found the iron(III) bisDHBS complex to produce an irreversible reductive peak associated with the Fe(III)/(II) reduction with a peak current at −670 mV vs Ag/AgCl 3 M NaCl, analogous to the one observed for iron(III) azotochelin at −660 mV vs Ag/AgCl 3 M NaCl.

The irreversible waveshapes recorded for iron(III) azotochelin and iron(III) bisDHBS are contrary to the near-reversible behavior reported for the iron(III) complexes of other catecholate and hydroxamate siderophores at pH 7.0, evaluated by the peak separation (Δ*E*) of cathodic (*E*_pc_) and anodic (*E*_pa_) peaks, as well as the ratio (*i*_pa_/*i*_pc_) of anodic (*i*_pa_) and cathodic (*i*_pc_) peak currents.^10,18,26,39^ The limited accounts of irreversible reductive voltammetry waveshapes attribute such electrochemical behavior to the presence of coordinatively unsaturated tetracoordinate iron(III) complexes, which upon reduction to their iron(II) counterparts are more prone to complex dissociation precluding the reoxidation of iron back to its 3+ oxidation state.^40^ Taylor et al. have investigated iron(III) complexes of simple catecholate (cat^2–^) ligands including [Fe(cat)_2_]^−^ through cyclic staircase voltammetry at a hanging MDE.^27^ The authors reported a voltammogram exhibiting only a reductive peak with an *E*_pc_ of −354 mV vs a saturated calomel electrode (SCE) (at pH 7.0) for the [Fe(cat)_2_]^−^ complex; UV–vis spectroscopy was employed to confirm the presence of a bis(catecholate) complex as indicated by its λ_max_ value of 570 nm. Spasojević et al. studied naturally occurring tetradentate siderophores bearing hydroxamate chelating groups using CV at gold and glassy carbon working electrodes.^26^ They have shown that alcaligin (Figure 1) forms the tetracoordinated species [Fe(alcaligin)(H_2_O)_2_]^+^ only in a sufficiently acidic environment (pH 2.0). Under these conditions, the complex exhibited an irreversible voltammetric wave with an *E*_pc_ shifted 400 mV positive of the value observed for the hexacoordinate Fe_2_(alcaligin)_3_ complex at pH 7.0. Similarly, Hou et al. performed UV–vis spectroscopic studies demonstrating the formation of the tetracoordinate complex of iron(III) alcaligin at pH 2.5 with a λ_max_ value of 472 nm, with the hexacoordinate species being predominant at physiologically relevant pH (λ_max_ = 426 nm; pH 6–9).^39^ The preference of dihydroxamate siderophores to form dimeric complexes with iron at neutral pH is attributed to the preference of iron(III) to exist in a six-coordinate environment, necessitating the low denticity siderophores to employ more than one ligand in order to saturate the metal coordination sphere. Such behavior is therefore expected from all low denticity siderophores; however, the irreversible reductive waveform we have observed for iron(III) azotochelin at pH 7.0 is not consistent with the existence of a tris(catecholate) complex with a coordinatively saturated iron(III) center.

### Characterization of Fe(III) Azotochelin

**Figure 5:**
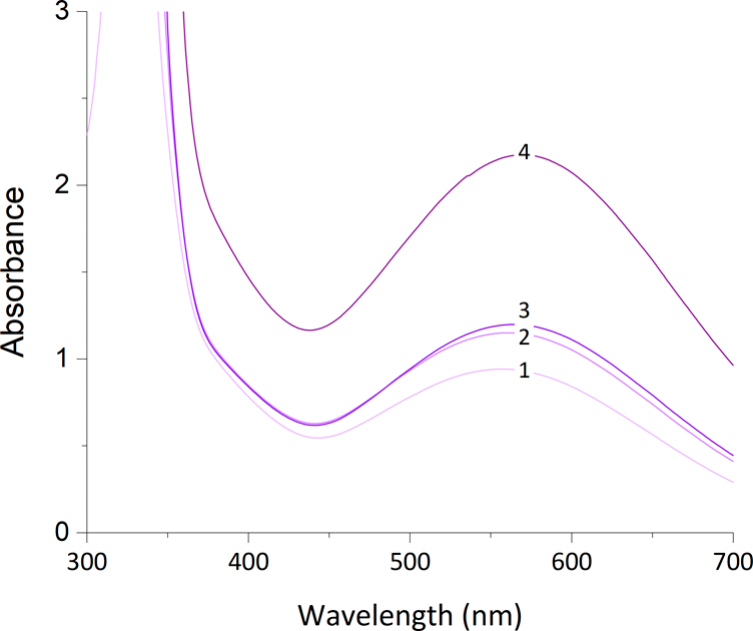
UV–vis absorption spectra of iron(III) azotochelin in a 5 mM BIS-TRIS buffer containing 100 mM NaCl at pH 7.0 as a function of varying M:L ratios: 1:1 (**1**), 1:2 (**2**), 1:3 (**3**), and 2:3 (**4**).

To elucidate the iron(III) coordination sphere of the species responsible for the irreversible voltammetric behavior exhibited by an iron(III) azotochelin solution at neutral pH, parallel UV–vis and native ESI MS structural studies were performed. Under conditions analogous to those used for the CV measurements (5 mM BIS-TRIS, 100 mM NaCl buffer solution at pH 7.0), the absorbance of iron(III) azotochelin at varying M:L ratios was measured after an equilibration period of 5 min following each addition (Figure 5). We obtained a λ_max_ value of 557 nm for a solution prepared in an equimolar M:L ratio, corresponding to the ligand-to-metal charge transfer band (LMCT) of iron(III) azotochelin. A small wavelength shift was observed upon the addition of 2 further equivalents of azotochelin, resulting in a λ_max_ of 563 nm for a sample in an M_1_:L_3_ ratio, with a further shift to a λ_max_ of 569 nm for an M_2_:L_3_ ratio solution. The small variations in the λ_max_ values across the different M:L ratios can be attributed to an equilibrium shift for the complexation of iron(III) by the siderophore, induced by changes in the concentration of individual species during the time course of the experiment. Our experimental values remain within the range characteristic for bis(catecholate) complexes as reported by Harris et al. and imply the preferable formation of a tetradentate iron(III) azotochelin under the conditions studied.^14^ Moreover, these results are in agreement with the λ_max_ of 566 ± 4 nm reported for iron(III) azotochelin at pH 7.0 in the literature.^35,36^ However, we question the assignment of this value by Cornish and Page to the presence of an iron(III) azotochelin complex in an M_2_:L_3_ ratio, as across the literature, iron(III) tris(catecholate) complexes characteristically exhibit a λ_max_ between 480 and 495 nm.^14,41,42^ This suggests that no significant changes in speciation accompany the solution ratio changes from a tetracoordinate M_1_:L_1_ complex to a hexacoordinate M_2_:L_3_ complex, as would be anticipated from the inherent preference of iron(III) to adopt octahedral coordination spheres.

Native ESI-MS was employed to obtain further evidence in support of the proposed formation of an iron(III) azotochelin M_1_:L_1_ complex at neutral pH, as implied by the irreversible voltammogram waveshapes obtained during solution CV, and the characteristic bis(catecholate) λ_max_ values from the UV–vis absorption spectra. The soft ionization approach utilized in native ESI-MS preserves noncovalent interactions of the analyte during the transition into the gas phase and has been previously successfully applied to the characterization of iron(III) complexes of catecholate siderophore-antibiotic conjugates.^7^ MS analysis requires the employment of a volatile buffer (ammonium acetate, NH_4_OAc) to enable the vaporization of the sample, and hence, CV and UV–vis experiments of iron(III) azotochelin were repeated under these conditions to validate the extent to which the differences in buffer compositions (BIS-TRIS versus NH_4_OAc) affect the iron(III) coordination sphere of the siderophore complex. Solution CV of iron(III) azotochelin in a 5 mM NH_4_OAc buffer (pH 7.0) containing 100 mM NaCl was performed at the BDD working electrode at varying M:L ratios (1:1, 1:2, 1:3, and 2:3) (Figure S4 and Table S2), and similar electrochemical behavior was observed to that recorded in the BIS-TRIS buffer (Figure S5). Similarly, an irreversible, one-electron reduction wave was detected at all M:L ratios with a maximum *E*_p_ shift of +30 mV in comparison to the reduction potential values measured in the BIS-TRIS buffer. This fluctuation in the *E*_p_ is attributed to the interaction of the positively charged ammonium ion with the negatively charged iron(III) azotochelin, [Fe^3+^Az^4–^]^−^, complex, an interaction that does not occur with the neutral BIS-TRIS molecules. We assume that the ionic pairing facilitates the reduction of iron(III) and thus shifts the *E*_p_ in the positive direction. However, this does not impose any changes on the first coordination sphere of the metal center and therefore allows a valid comparison between the MS, CV, and UV–vis data. Prior to MS characterization, the coordination of iron by the siderophore ligand was verified with UV–vis absorption spectroscopy in a 10 mM NH_4_OAc buffer (pH 7.0) for all the M:L ratios analyzed (1:1 and 2:3) (Figure S6).

MS in the negative ionization mode revealed a peak at *m*/*z* 470.0425 for samples prepared in both M_1_:L_1_ and M_2_:L_3_ ratios (Figure S7), which we can attribute to the presence of a coordinatively unsaturated tetracoordinate M_1_:L_1_ species of composition [Fe^3+^Az^4–^]^−^. No evidence was obtained for the formation of a coordinatively saturated hexacoordinate M_2_:L_3_ complex by iron(III) azotochelin, [(Fe^3+^)_2_(Az^4–^)_3_]^6–^, at either of the M:L ratios analyzed. Interestingly, in the positive ionization mode (Figure S8), peaks at *m*/*z* 472.0583 in the M_1_:L_1_ spectrum and 472.0573 for the M_2_:L_3_ sample were detected. Both these peaks exhibit an approximate 2.0 *m*/*z* increase in comparison to the molecular ion peak detected in the negative ionization mode. From the +1 charge of these peaks, this increase in *m*/*z* can be attributed to the addition of 2H^+^ ions to the [Fe^3+^Az^4–^]^−^ complex at both M:L ratios, [(Fe^3+^Az^4–^)^−^ + 2H^+^]^+^. As described above, under sufficiently acidic conditions it has been shown that the protonation of the two meta catechol oxygens (Figure 2) in catecholamide siderophores results in a switch from catecholate- to salicylate-type bonding with no change in complex stoichiometry.^43^ The occurrence of this phenomenon would allow the assignment of the observed peak in both spectra to [Fe^3+^H_2_Az^2–^]^+^, a salicylate-coordinated iron(III) azotochelin complex in an M_1_:L_1_ ratio, in agreement with the p*K*_a_ values reported for the meta phenolic oxygens of azotochelin, log *K*_4_ = 7.41(3) and log *K*_3_ = 8.54(4).^44^ However, it is important to note that while the ESI-MS data suggests the existence of two species in equilibrium, this is not supported by the CVs which imply a one-electron transfer under the conditions studied. A recent report in the literature has highlighted the high pH lability of ammonium acetate buffer, noting that under the conditions of a positive mode in native ESI-MS, NH_4_OAc is prone to undergoing acidification, effectively lowering the pH to 4.75 ± 1.^45^ Thus, the MS spectrum in positive ionization mode is unlikely to reflect the true composition of the solution at pH 7.0. Nonetheless, the remainder of the data allows a confident assignment of the irreversible reduction peak at −670 ± 10 mV vs Ag/AgCl 3 M NaCl to the iron(III) reduction of a tetracoordinated [Fe^3+^Az^4–^]^−^ complex, the dominant species in solution at pH 7.0.

As described above, the preference for iron(III) azotochelin to adopt a coordinatively unsaturated configuration is unusual in comparison to the iron complexes of bis(hydroxamate) siderophores. However, similar behavior has been observed with amonabactin and chrysobactin, bis- and monocatecholamide siderophores, respectively, implying a high dependency of complex stoichiometry on the nature of coordinating groups in low denticity siderophores which has not been explicitly noted in the literature before.^29,46^ The high electron density on the oxygen donor atoms, promoted through the resonance stabilization of the neighboring aromatic ring, increases the affinity of catechol groups toward iron(III) as opposed to hydroxamate-based siderophores, where the resonance stabilization arises from the nitrogen lone pair and hence is less extensive.^47^ Subsequently, it can be proposed that the higher affinity of catechol groups toward iron(III) allows for the formation of coordinatively unsaturated complexes of catecholamide siderophores.

The formation of a tetracoordinate iron(III) azotochelin species is consistent with the observation of an irreversible waveshape in the CV. Upon the reduction of the iron(III) center to iron(II), the thermodynamic stability of the tetracoordinate metal siderophore complex is significantly lowered, and its lability increased, resulting in the dissociation of the complex on the electrochemical time scale, subsequently preventing the reoxidation of the iron center. Unfortunately, experiments at higher scan rates (50, 100, and 500 mV s^–1^) were unsuccessful in detecting the oxidation of the iron(II) center prior to the dissociation of the complex, predominantly due to the large capacitive current observed. It is noteworthy that electrochemical investigations of iron(III) chrysobactin were able to detect both cathodic and anodic peaks; however, in these experiments, a static mercury drop working electrode was employed.^21^ The authors highlighted the affinity of mercury toward the catechol groups which has promoted the absorption of the complex onto the electrode surface, as indicated from a linear peak current (*i*_pc_) vs scan rate (*v)* dependence. This was proposed to have a stabilization effect on the iron(III) complex, subsequently preventing its dissociation and leading to a reversible cyclic voltammogram.

### The Effect of pH and Buffer on the Electrochemical Behavior of Fe(III) Azotochelin

The stability of iron(III) siderophore complexes and subsequently their reduction potentials are strongly dependent on the pH.^10,26^ As the chelation of the metal ion requires deprotonation of the coordinating donor atoms, increased competition between iron(III) and H^+^ ions is observed at lower pH. Iron(III) siderophore complexes adapt to pH fluctuations through the reorganization of the metal’s coordination sphere, and these adaptations are controlled by the siderophore structure. As described earlier, low-denticity hydroxamate siderophores exhibit changes in the stoichiometry of their iron(III) complexes, whereas catecholamide siderophores are capable of undergoing shifts in their coordination mode. Azotochelin is a low denticity catecholamide siderophore, and hence, it is unknown which strategy it employs as pH electrochemical studies on iron bis(catecholate) siderophore complexes have not been previously reported.

**Figure 6:**
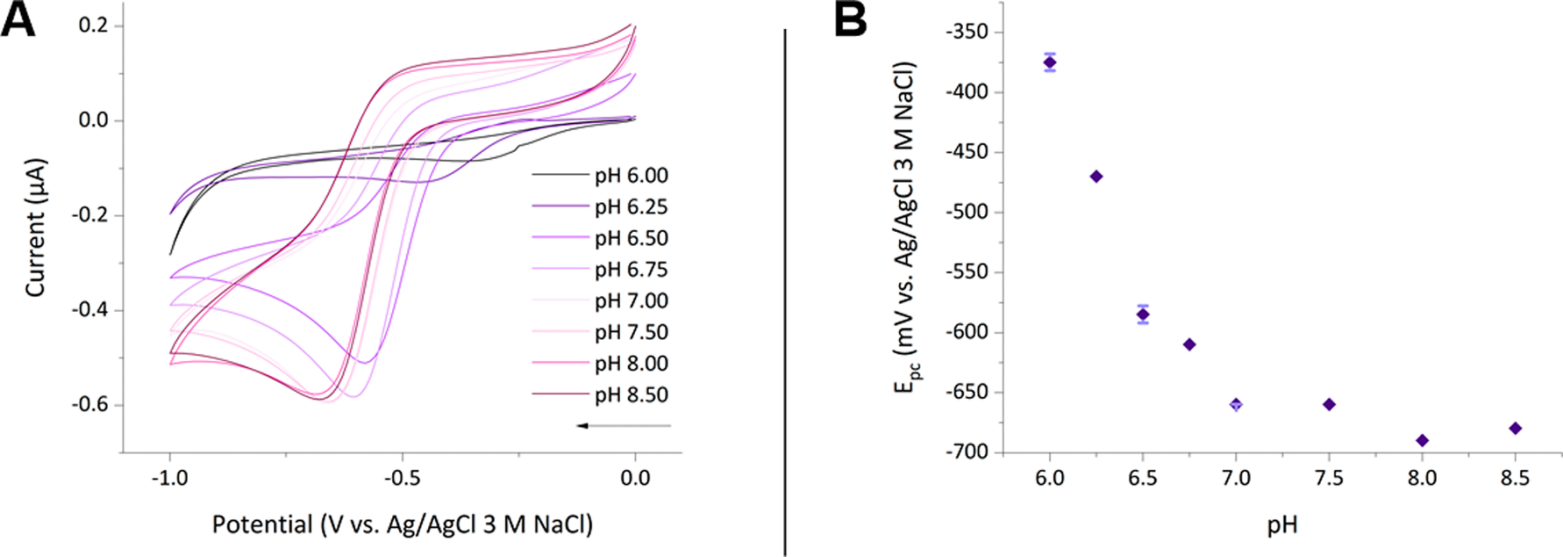
(**A**) Cyclic voltammograms of iron(III) azotochelin (M_1_:L_1_) in a 5 mM BIS-TRIS buffer, 100 mM NaCl at pH 7.0. Analyte concentrations of [Fe] = 0.45 mM and [Az] = 0.45 mM; *v* = 10 mV s^–1^, *E*_step_ = 0.01 V. The arrow indicates the direction of the current. (**B**) The pH dependence of the reduction potential (*E*_p_) for Fe(III) azotochelin, extracted from CVs in (A). SD error bars are shown for pH 6.0, 6.5, and 7.0.

The electrochemical examination of iron(III) azotochelin was expanded to cover a biologically relevant pH range of 6.0–8.5, by recording CVs at 0.25 and 0.5 pH increments, utilizing a 5 mM BIS-TRIS buffer containing 100 mM NaCl as the supporting electrolyte (Figure 6). The same experimental procedure was employed as in the initial investigation at pH 7.0 where the CVs were measured after sequential additions of each stock solution, following an equilibration period of 5 min after each step. A single irreversible waveshape was recorded at all pH values for an equimolar ratio of M:L (Figure 6A), implying the M_1_:L_1_ tetracoordinate complex was preserved under all experimental conditions. The same electrochemical irreversibility was observed regardless of the M:L ratio employed. No notable changes in the *E*_pc_ were detected above pH 7.0 with a maximum shift of −30 mV (pH 8.0), which indicates the stability of the iron(III) azotochelin complex under these conditions. The small *E*_p_ shift can be attributed to the decreased proton concentration and subsequently reduced competition between iron(III) and H^+^ ions. Disparate behavior was recorded under acidic conditions, with an initial positive shift in the reductive peak potential of 40 mV and 80 mV at pH 6.5 and 6.75, respectively, as well as a stepwise decrease in the Faradaic current. Lowering the pH further to 6.0 induced a significant *E*_p_ shift of +290 mV and an 86% decrease in the *i*_pc_ in comparison to the CV recorded at pH 7.0, implying a change to the metal coordination sphere which has resulted in an iron(III) azotochelin complex of lower stability. The decrease in the Faradaic current could be consistent with a change in the coordination mode, which causes the overall charge of the complex to change from −1 in the catecholate-coordinated complex [Fe^3+^Az^4–^]^−^ to +1 in the salicylate-coordinated complex [Fe^3+^H_2_Az^2–^]^+^, resulting in electrostatic repulsion between the salicylate-coordinated complex and the positively charged H-terminated surface of the BDD electrode.^48^

**Figure 7:**
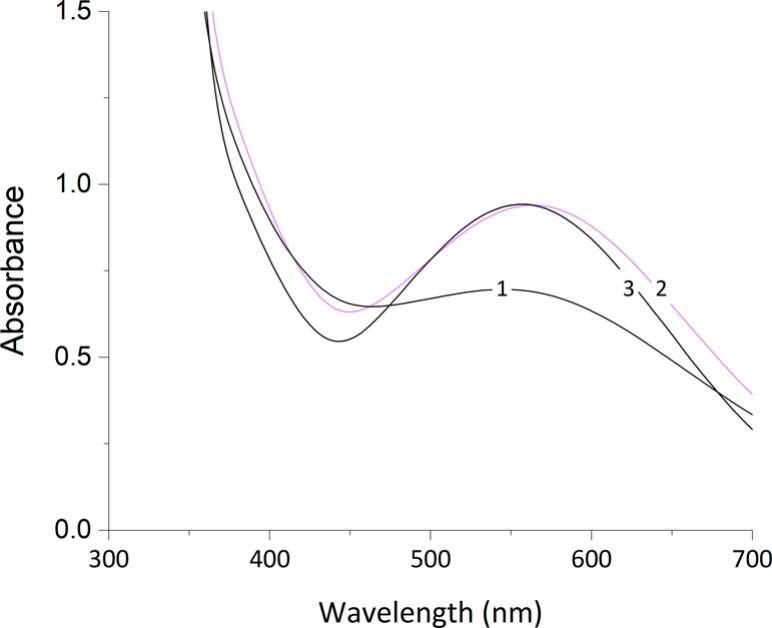
UV–vis absorption spectra of iron(III) azotochelin in a 5 mM BIS-TRIS buffer containing 100 mM NaCl as a function of pH: 6.0 (**1**), 6.5 (**2**), and 7.0 (**3**). Analyte concentrations of [Fe] = 0.45 mM and [Az] = 0.45 mM.

To further examine the proposed change in the coordination mode, a complementary UV–vis study on the impact of pH on the coordination chemistry of iron(III) azotochelin was performed. As before, the measurements were conducted under conditions and procedures identical to those employed in the electrochemical investigation. The position of the λ_max_ of the LMCT band remained relatively unchanged (555 ± 9 nm) at all pH values investigated (Figure 7), implying that the M_1_:L_1_ stoichiometry of the iron(III) azotochelin complex persisted even at lower pH. However, a decrease in the absorbance of the LMCT band was observed at pH 6.0, indicating a lower concentration of the species containing the iron(III)-catecholate chromophore. This supports the formation of a secondary species at the expense of a catecholate-coordinated iron(III) azotochelin complex resulting in a mixture of the two species. As described in the literature, these results imply a shift in the coordination mode from catecholate to salicylate as the latter lacks the iron-catechol chromophore responsible for the LMCT band.

In addition, ESI-MS was employed to characterize the species under investigation and confirm the formation of a salicylate-coordinated iron(III) azotochelin complex in acidic conditions. Mass spectra (Figure S9) obtained at pH 6.0 (and pH 6.5) reveal the presence of peaks at *m*/*z* 470.0423 (470.0425) in the negative ionization mode and *m*/*z* 472.0578 (472.0570) in the positive ionization mode (Figure S10) which correspond to the presence of both catecholate-coordinated [Fe^3+^Az^4–^]^−^ and salicylate-coordinated [Fe^3+^H_2_Az^2–^]^+^ complexes, respectively. Similar to the mass spectrometric measurements performed at pH 7.0, no peaks associated with hexacoordinate complexes or any of their related adducts were detected. As noted previously, the NH_4_OAc buffer undergoes acidification in the positive ionization mode, effectively lowering the pH of the solution, and thus, these results cannot be viewed as an accurate depiction of iron complexation at pH 6.0 or 6.5. However, it can be used as supplementary evidence to the more conclusive UV–vis and CV data that indicate that as the pH of the solution is lowered below pH 7.0, the concentration of the less thermodynamically stable salicylate-coordinated complex increases, resulting in a positive *E*_p_ shift. At pH below 6.25, [Fe^3+^H_2_Az^2–^]^+^ becomes the dominant species in solution as implied by a large shift in *E*_p_ of 290 mV and a drastic decrease in the Faradaic current. The latter is due to the electrostatic repulsion between the salicylate-coordinated complex and the positively charged H-terminated surface of the BDD electrode, as described above.

The preference of iron(III) azotochelin to form a tetracoordinated bis(catecholamide) complex over a hexacoordinated tris(catecholamide) species, as demonstrated above, results in two unoccupied coordination sites at the metal center that are available for binding of secondary molecules. We have been inspired by literature reports on the effects of buffer composition on the *E*_p_ of coordinatively saturated iron(III) siderophore complexes to investigate variations in *E*_p_ of iron(III) azotochelin in the presence of different buffers at pH 7.0. The results are summarized in Table 1, and the additional commentary as well as the corresponding CVs are available in the SI (Figures S11, S12).

**Table 1:**
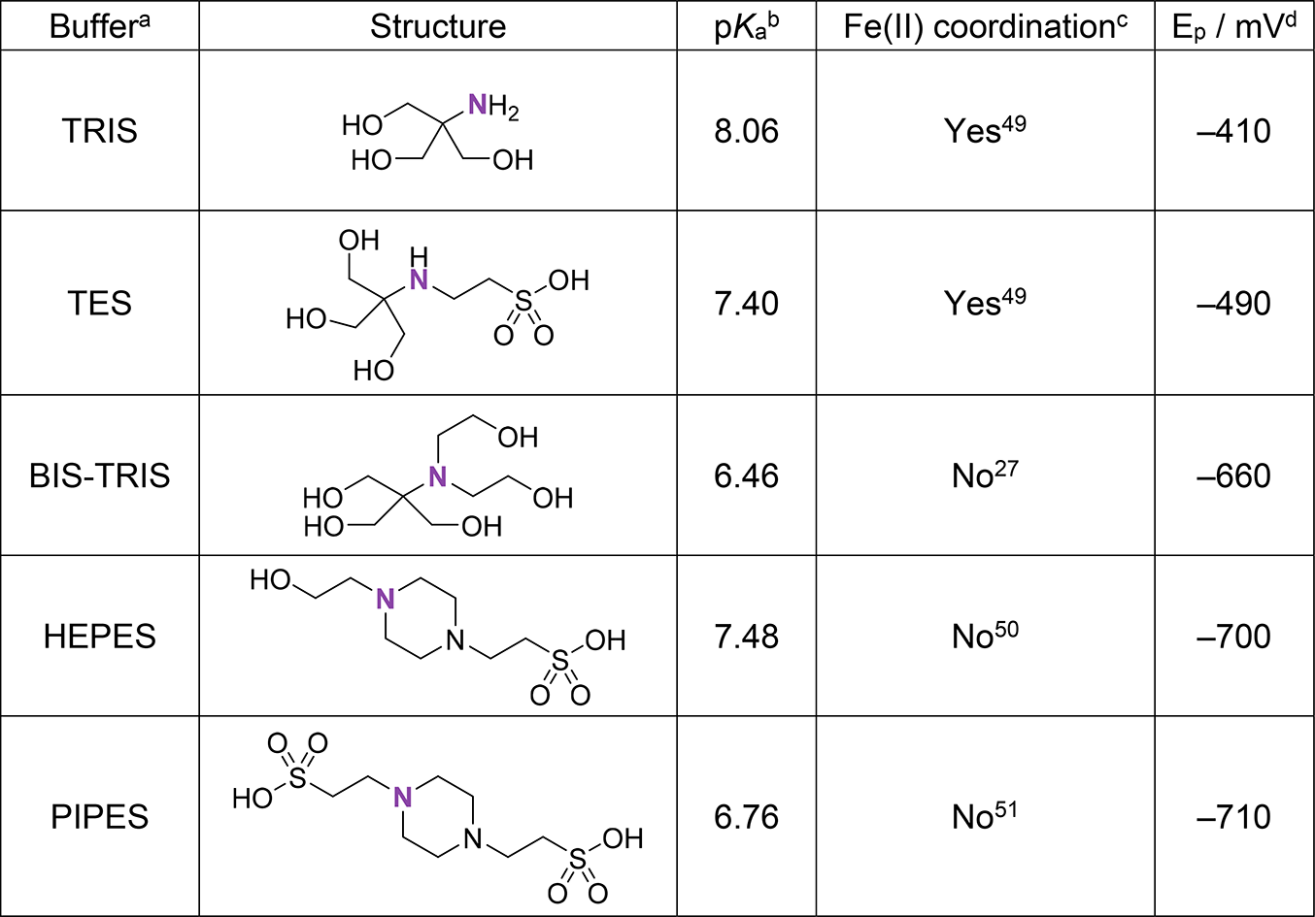
Dependence of Iron(III) Azotochelin Reduction Potential (*E*_p_) on the Type of Buffer Salt Employed in Aqueous Solution Cyclic Voltammetry

a TRIS = 2-amino-2-(hydroxymethyl)-1,3-propanediol, TES = *N*-[tris(hydroxymethyl)methyl]-2-aminoethanesulfonic acid, BIS-TRIS = bis(2-hydroxyethyl)amino-tris(hydroxymethyl)methane, HEPES = 4-(2-hydroxyethyl)piperazine-1-ethanesulfonic acid, PIPES = piperazine-1,4-bis(2-ethanesulfonic acid).

b At 25 °C.^52^

c This column summarizes the most prevalent findings from the literature; however, it is to be noted that these strongly depend on the experimental conditions, and hence, discrepancies may be present between reports. Therefore, it is suggested the information should only be referred to as a guideline.

d *E*_p_ vs Ag/AgCl 3 M NaCl; 5 mM buffer salt, 100 mM NaCl; pH 7.0; analyte concentrations of [Fe] = 0.45 mM and [Az] = 0.45 mM; *v* = 10 mV s^–1^, *E*_step_ = 0.01 V.

Most notably, versus the BIS-TRIS buffer an *E*_p_ shift of +170 mV and +250 mV was observed for an equimolar solution of iron(III) azotochelin in TES and TRIS buffers (Figure S11), respectively, indicating a decrease in the stability of the redox-active iron(III) species. Both buffers have been described in the literature to exhibit metal-coordinating capabilities, presumably through the primary and secondary nitrogen atoms. The coordination of a soft/intermediate donor atom, such as nitrogen, stabilizes the iron(II) relative to the iron(III) azotochelin complex, facilitating the addition of an electron to the metal center and subsequently increasing the reduction potential of the complex.

The relationship between the reduction potential of the redox center and the coordination environment points toward a direct interaction of the iron(III) center with buffer molecules. This highlights the importance of experimental conditions, particularly buffer compositions, when designing experiments and making comparisons between the reduction potential values of iron(III) siderophores across the literature. From the above study, the BIS-TRIS buffer emerges as the best choice for future studies due to the minimal interaction with the redox-active iron-center, particularly in the presence of free coordination sites on the metal center.

### Electrochemical Investigation of Hexadentate Siderophores

To extend our investigation into the use of BDD as the working electrode for the measurement of the Fe(III)/(II) redox couple of iron(III) siderophores, we electrochemically investigated two tris(catecholate) siderophores, protochelin and enterobactin, and a tris(hydroxamate) siderophore, ferricrocin. The same experimental protocol as the one used for bis(catecholate) siderophores was employed.

**Figure 8:**
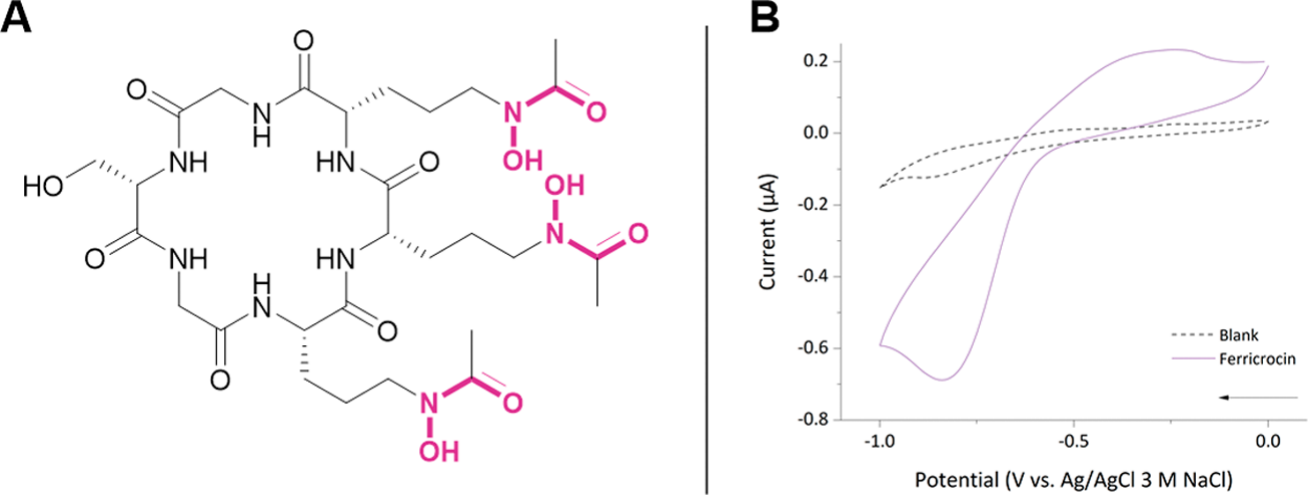
(**A**) Chemical structure of ferricrocin, a tris(hydroxamate) siderophore with its iron binding units highlighted in pink. (**B**) Cyclic voltammogram of a ferricrocin solution in a 5 mM BIS-TRIS buffer containing 100 mM NaCl at pH 7.0. Analyte concentrations of [Ferr] = 0.45 mM; *v* = 10 mV s^–1^, *E*_step_ = 0.01 V. The arrow indicates the direction of the current.

Regrettably, the reduction potential for the two hexadentate catecholate siderophores through CV measurements (Figure S13) on a BDD working electrode was not accessible. An increase in the cathodic current in comparison to the CV of the blank buffer solution was observed for iron(III) enterobactin at potentials of ca. >−600 mV (pH 7.0) (Figure S13B); however, the maximum of the reductive peak was beyond the voltage window accessible to the BDD electrode, highlighting the limited usability of this electrode material for iron(III) complexes of tris(catecholate) siderophores with highly negative reduction potentials. In contrast, the CV of ferricrocin (Figure 8) exhibits a quasi-reversible waveshape, displaying both the reduction and oxidation peaks associated with the Fe(III)/(II) redox couple, albeit with a large Δ*E* of 600 mV. The detection of the anodic current (unobserved in the electrochemistry of iron(III) azotochelin and iron(III) bisDHBS) is most likely due to the preorganized architecture of the cyclic hexadentate siderophore, and the formation of a coordinatively saturated iron(III) complex, minimizing the dissociation of the iron(II) siderophore complex on the electrochemical time scale. At the BDD working electrode, the formal midpoint potential, *E*_1/2_, was observed at −540 mV vs Ag/AgCl 3 M NaCl (−350 mV vs NHE), which is slightly more negative than the *E*_1/2_ value of −412 mV vs NHE (Δ*E* 60–69 mV) reported by Wong et al. The latter was measured at a hanging MDE in 100 mM phosphate, 1 M KCl buffer (pH 8.0).^53^ We attribute these differences in the *E*_1/2_ values and the extent of electrochemical reversibility to the slower electron transfer kinetics observed at the BDD electrode, supported by our control experiments that examined the electrochemical behavior of ferricyanide at the BDD electrode (Tables S2 and S3). Moreover, as demonstrated above, the electrochemical behavior of siderophore complexes is strongly dependent on the solution composition, and the differences in buffer choice and pH will have contributed to the differences observed.

## Summary and Conclusions

This study reports for the first time an electrochemical investigation of iron(III) azotochelin, a tetradentate bis(catecholate) siderophore. Examining the redox behavior of siderophore complexes is crucial for understanding their role in the siderophore-mediated iron transfer in microorganisms, particularly the intracellular release of iron. Cyclic voltammetry revealed an irreversible reductive waveshape with a peak potential of −660 mV vs Ag/AgCl 3 M NaCl (−470 mV vs NHE), corresponding to the Fe(III)/(II) redox couple. A complementary UV–vis and native ESI-MS analysis provided evidence for the formation of a coordinatively unsaturated (M_1_:L_1_) iron(III) azotochelin complex. The inclination of the bis(catecholate) siderophore to form a tetracoordinate complex is different from that of tetradentate bis(hydroxamate) siderophores which form M_2_:L_3_ complexes and exhibit reversible cyclic voltammograms at pH 7.0 but with reduction potentials similar to that of iron(III) azotochelin. We propose this difference in behavior to be associated with the higher affinity of catechols toward the iron metal center in comparison to the hydroxamate ligands as this can compensate for the lower stability arising from the incomplete coordination sphere.

The investigation of the iron(III) azotochelin electrochemical behavior at biologically relevant pH values (6.0–8.5) revealed a significant positive *E*_p_ shift of 290 mV upon acidification from pH 7.0 to pH 6.0, which upon supplementation with UV–vis and native ESI-MS data was proposed to result from a switch from catecholate- to salicylate-type coordination around the metal center. Moreover, a positive *E*_p_ shift of 250 mV and 170 mV was measured in buffers comprising primary and secondary amines, as exemplified by TRIS and TES, respectively, in comparison to the reduction potential measured in BIS-TRIS which contains a nonmetal coordinating tertiary amine. This is hypothesized to arise from the coordination of the soft/intermediate nitrogen to the iron center resulting in the stabilization of the iron(II) over the iron(III) complex. While this could provide an insight into the role of ternary complexes in the intracellular redox release mechanism of iron, we believe that more importantly it signifies that caution should be employed when making comparisons between the reduction potential of iron(III) siderophores across different reports as these might heavily depend on the conditions employed.

In conclusion, we have demonstrated that electrochemical investigations of iron(III) azotochelin complexes can be carried out at BDD working electrodes, with excellent reproducibility and sensitivity, demonstrating its feasibility to replace mercury-based electrodes in the electrochemical studies of low denticity catecholate siderophores and high denticity hydroxamate siderophores. We believe our findings can also be extended to low denticity hydroxamate siderophores as they exhibit similar or more positive reduction potential values than the complexes we have studied. We hope this will prove to be of interest not only to researchers working on the discovery of new siderophores but also to those hoping to utilize siderophores in alternative applications, where the capacity for the modulation of the complex’s reduction potential through changes in the metal coordination sphere can be beneficial in creating redox-responsive systems.

## Experimental Section

### General Remarks

All chemical reagents and solvents were obtained from commercial suppliers and used as supplied. When required, solvents were either dried over activated 4 Å molecular sieves or obtained from a Prosolv MD 7 solvent purification system, which involves the passage of the solvent through two columns packed with molecular sieves. Flash column chromatography was performed on silica gel (60 Å pore size, 220–440 mesh particle, 35–75 μm) as a stationary phase, and elution was achieved with the appropriate solvent systems. Reactions were visualized on Merck silica gel 60 F254 aluminum backed plates using a UVItec LF-204.S lamp or stained with potassium permanganate. Characterization was achieved with NMR and HRMS. NMR spectra were recorded on a Joel ECS 400 MHz instrument: ^1^H NMR at 400 MHz and ^13^C NMR at 101 MHz. All NMR assignments were supported with COSY, DEPT-135, and HMQC experiments where required. High-resolution mass spectra (HRMS) were recorded using the electrospray ionization (ESI) technique on a Bruker compact TOF electrospray mass spectrometer by either Karl Heaton or Angelo Lopez. Native ESI-MS studies were performed on the same instrument by Karl Heaton. UV–vis measurements were recorded on a UV-1800 Shimadzu spectrophotometer using Starna Scientific quartz cuvettes.

### Cyclic Voltammetry (CV)

The voltammograms were recorded on a computer-controlled PalmSens4 potentiostat and the associated PSTrace software. All measurements were performed at room temperature in an anaerobic glovebox maintained under a nitrogen atmosphere (<4 ppm of O_2_) in a Reacti-Vial (Thermo Scientific) as the electrochemical cell. A three-electrode setup was employed comprising of a 3 mm diameter boron-doped diamond working electrode (BioLogic)^55^, an Ag/AgCl 3 M NaCl reference electrode (BASi), and a platinum wire auxiliary electrode (in-house). The working electrode was polished using sonication in Milli-Q H_2_O (3 × 15 s) prior to taking the measurements. Electrochemical characterization of the BDD working electrode using the ferri/ferrocyanide redox couple as well as the conversion between the Ag/AgCl reference electrode and the normal hydrogen electrode (NHE) is included in the Supporting Information. Unless otherwise stated, the CV experiments were performed within a potential window from 0.0 V to −1.0 V vs Ag/AgCl 3 M NaCl at a scan rate of 10 mV s^–1^ and a potential step of 0.01 V, the third scan was recorded. [Experiments within individual studies were carried out within short timeframes of several days, and hence, the difference in capacitance between the voltammograms can be attributed to changes in the solution rather than variations in the surface of the BDD electrode. It is worthy to highlight that we have observed the deterioration of the BDD surface over longer time scales (6–8 months) which has resulted in the broadness of cathodic peaks and prevented accurate determination of the maximum peak potential. We believe this is due to the oxidation of the electrode surface resulting in a change to surface termination from C–H^δ+^ to C–O^δ-^.^55^]

### Synthesis

*N*^2^,*N*^6^-Bis(2,3-dihydroxybenzoyl)-*L*-lysine (**azotochelin**): *N*^2^,*N*^6^-bis(2,3-dihydroxybenzoyl)-*L*-lysine was synthesized from *L*-lysine monochloride and 2,3-bis(benzyloxy)benzoic acid-*N*-hydroxysuccinimide ester, followed by hydrogenation to remove the benzyl protecting groups, as previously reported by Chimiak and Neilands.^37^

^**1**^**H NMR:** (400 MHz, MeOH-*d*_4_): δ 7.30 (d, *J* = 8.0 Hz, 1H, Ar-H), 7.15 (d, *J* = 8.1 Hz, 1H, Ar-H), 6.90 (dd, *J* = 12.2, 7.7 Hz, 2H, Ar-H), 6.68 (dt, *J* = 15.4, 8.0 Hz, 2H, Ar-H), 4.60 (dd, *J* = 8.9, 5.0 Hz, 1H, NH-CH-CO), 3.37 (t, *J* = 7.0 Hz, 2H, NH-CH_2_), 2.06–1.98 (m, 1H, *CH_2_-CH), 1.94–1.85 (m, 1H, *CH_2_-CH), 1.73–1.61 (m, 2H, NH–CH_2_–CH_2_), 1.57–1.47 (m, 2H, CH_2_–CH_2_-*CH_2_).

^**13**^**C NMR:** (101 MHz, MeOH-*d*_4_): 174.74, 171.54, 170.91, 150.33, 149.83, 147.31, 147.22, 119.75, 119.54, 119.48, 119.59, 53.79, 40.20, 32.25, 29.99, 24.41.

**HRMS: (ESI):** Calcd for C_20_H_21_N_2_O_8_ [M – H]^−^ 417.1303; found 417.1305.

*N*^1^-[*N*^2^,*N*^6^-Bis(2,3-dihydroxybenzoyl)-*L*-lysyl]-*N*^4^-(2,3-dihydroxybenzoyl)-1,4-diamino-butane (**protochelin**): *N*^1^-[*N*^2^,*N*^6^-bis(2,3-dihydroxybenzoyl)-*L*-lysyl]-*N*^4^-(2,3-dihydroxy-benzoyl)-1,4-diaminobutane was synthesized from *N*^2^,*N*^6^-bis(2,3-dibenzyloxybenzoyl)-*L*-lysine and (2,3-dibenzyloxybenzoyl)-diaminobutane hydrochloride, followed by hydrogenation to remove the benzyl protecting groups, as previously reported by Duhme et al.^54^

^**1**^**H NMR:** (400 MHz, MeOH-*d*_4_): δ 7.31 (dd, *J* = 1.53, 8.09 Hz, 1H, Ar-H), 7.21–7.17 (m, 2H, Ar-H), 6.94–6.89 (m, 3H, Ar-H), 6.73–6.66 (m, 3H, Ar-H), 4.54–4.51 (m, 1H, CH), 3.40–3.34 (m, 4H, NH-CH_2_-CH_2_), 3.27–3.22 (m, 2H, CH_2_-NH-C=O), 1.95–1.79 (m, 2H, CH_2_-CH), 1.69–1.54 (m, 8H, CH_2_–CH_2_-CH_2_).

^**13**^**C NMR:** (101 MHz, MeOH-*d*_4_): δ 210.11, 174.38, 171.52, 170.78. 149.50, 147.32, 147.17, 119.80, 119.69, 119.54, 118.59, 117.26, 116.71, 55.05, 40.14, 40.05, 32.98, 30.67, 30.05, 27.78, 24.38.

**HRMS: (ESI):** Calcd for C_31_H_35_N_4_O_10_ [M – H]^−^ 623.2359; found 623.2376.

## Supporting Information Available

Additional cyclic voltammetry data for iron(III) azotochelin for other M:L ratios studied in BIS-TRIS, chemical structure and CV of iron(III) bisDHBS; cyclic voltammograms of iron(III) azotochelin in TES, TRIS, PIPES, and HEPES buffers; UV–vis characterization of MS samples and all native ESI-MS spectra; cyclic voltammograms for ferric complexes of protochelin and enterobactin; electrochemical characterization of BDD working electrode; and determination of conversion factor for the reference electrode (PDF)
