# Supplementary material for: Electrochemical and Solution Structural Characterization of Fe(III) Azotochelin Complexes: Examining the Coordination Behavior of a Tetradentate Siderophore
Source: Inorg Chem. 2022 Oct 17;61(48):19172–82. doi: 10.1021/acs.inorgchem.2c02777 (PMC9727729; doi:10.1021/acs.inorgchem.2c02777)
Supplement: Supplementary file 1 — ic2c02777_si_001.pdf [file ic2c02777_si_001.pdf]

## Supporting information for:

# Electrochemical and solution structural characterisation of Fe(III) azotochelin complexes: Examining the coordination behaviour of a tetradentate siderophore

*Natalia G. Baranska, Alison Parkin* and Anne-Kathrin Duhme-Klair**

Department of Chemistry, University of York, Heslington, York, YO10 5DD, United Kingdom

***E-mail:** alison.parkin@york.ac.uk; anne.duhme-klair@york.ac.uk

**CONTENT**

CV characterisation data for Fe(III) azotochelin and Fe(III) bisDHBS………….. S2

Native ESI-MS studies of Fe(III) azotochelin……………………………………… S5

Buffer study data for Fe(III) azotochelin………………..………………………….. S12

CVs of Fe(III) tris(catecholate) complexes……………………………………..... S14

BDD working electrode characterisation…………………………………...…… S15

Reference electrode conversion……………………………………………………. S16

References……………………………………………………………………………. S16

## CV characterisation data for Fe(III) azotochelin and Fe(III) bisDHBS.

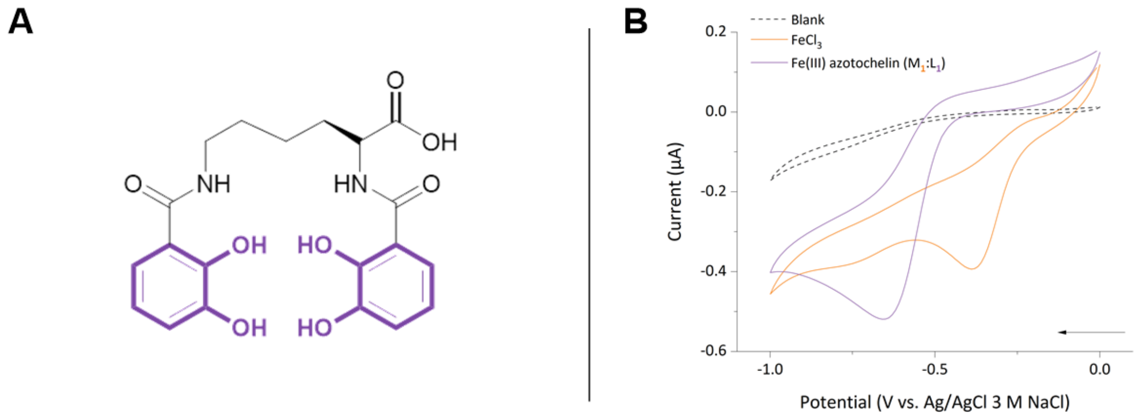


**Figure S1.** (**A**) Chemical structure of azotochelin. (**B**) Sequential cyclic voltammograms for the in-situ formation of an equimolar ($M_1$:$L_1$) Fe(III) azotochelin solution in 5 mM BIS-TRIS buffer containing 100 mM NaCl at pH 7.0; an aqueous iron stock (10 mM $FeCl_3$) was added to the buffer solution, following an addition of siderophore stock (10 mM azotochelin) in DMSO. Final analyte concentrations [Fe] = 0.47 mM, [Fe] : [Az] = 0.45 mM; $v$ = 10 mV $s^{-1}$, $E_{step}$ = 0.01

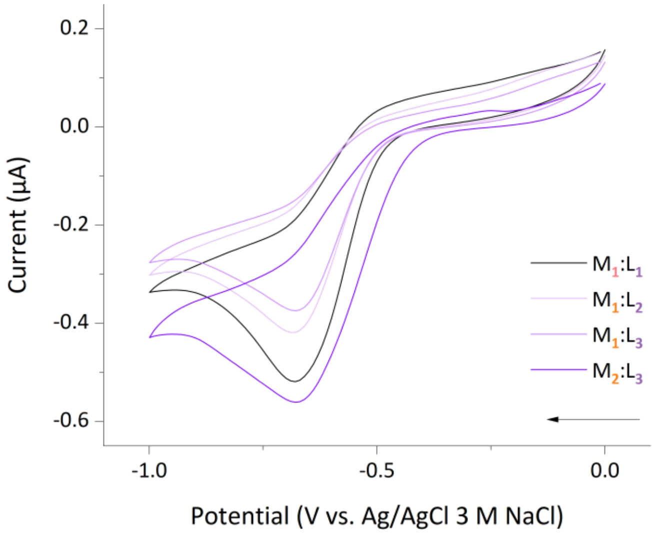


**Figure S2.** Cyclic voltammograms of a Fe(III) azotochelin solution in 5 mM BIS-TRIS buffer containing 100 mM NaCl at pH 7.0 at different ratios of metal (M) to ligand (L). $M_1$:$L_1$ = [Fe] = 0.45 mM, [Az] = 0.45 mM; $M_1$:$L_2$ = [Fe] = 0.43 mM, [Az] = 0.87 mM; $M_1$:$L_3$ = [Fe] = 0.42 mM, [Az] = 1.25 mM; $M_2$:$L_3$ = [Fe] = 0.80 mM, [Az] = 1.20 mM. $v$ = 10 mV $s^{-1}$, $E_{step}$ = 0.01 V. The arrow indicates the direction of the current.

**Table S1.** The tabulated reduction potential ($E_p$ vs Ag/AgCl 3 M NaCl) values and the corresponding peak currents ($i_p$) extracted from the cyclic voltammograms of different M:L ratio Fe(III) azotochelin solutions in BIS-TRIS buffer at pH 7.0.

| M:L | [Fe] / mM | [Az] / mM | $E_p$ / mV[a] | $-i_p$ / μA |
|---|---|---|---|---|
| 1:1 | 0.45 | 0.45 | –660 | 0.596 |
| 1:2 | 0.43 | 0.87 | –670 | 0.472 |
| 1:3 | 0.42 | 1.25 | –670 | 0.397 |
| 2:3 | 0.80 | 1.20 | –680 | 0.556 |

[a] $E_p$ vs Ag/AgCl 3 M NaCl; 5 mM BIS-TRIS buffer, 100 mM NaCl, pH 7.0; $v$ = 10 mV $s^{-1}$; $E_{step}$ = 0.01 V.

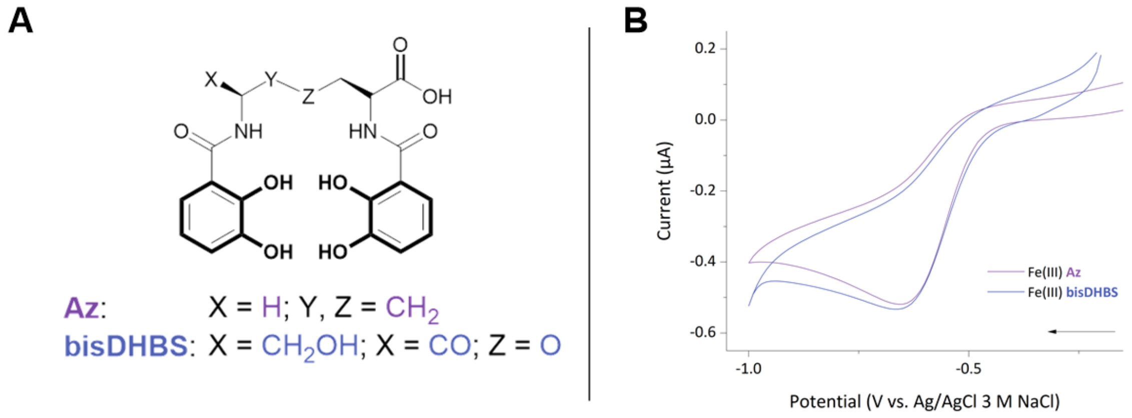


**Figure S3.** (**A**) Comparison of chemical structures between the two bis(catecholate) siderophores, azotochelin (Az) and bis(2,3-dihydroxybenzoyl-L-serine) (bisDHBS). (**B**) Cyclic voltammograms of an equimolar ($M_1$:$L_1$) iron(III) Az and iron(III) bisDHBS solutions in 5 mM BIS-TRIS buffer containing 100 mM NaCl at pH 7.0. Analyte concentrations of [Fe] = 0.45 mM; [siderophore] = 0.45 mM; v = 10 mV $s^{-1}$, $E_{step}$ = 0.01 V. The arrow indicates the direction of the current.

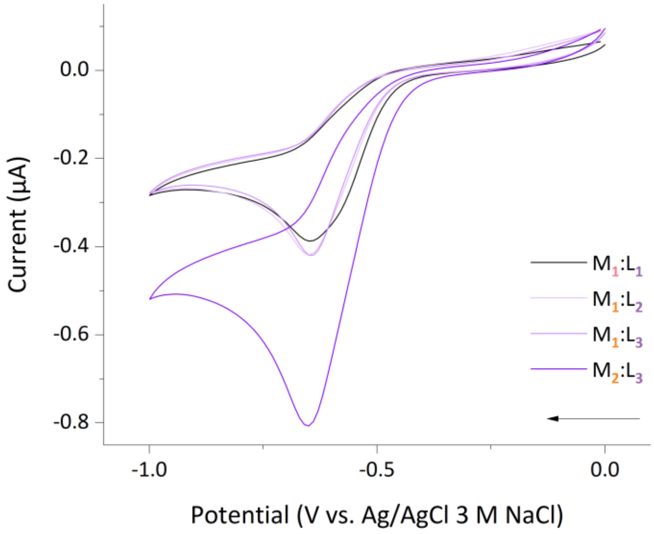


**Figure S4.** Cyclic voltammograms of a Fe(III) azotochelin solution in 5 mM $NH_4OAc$ buffer containing 100 mM NaCl at pH 7.0 at different ratios of metal (M) to ligand (L). $M_1:L_1$ = [Fe] = 0.45 mM, [Az] = 0.45 mM; $M_1:L_2$ = [Fe] = 0.43 mM, [Az] = 0.87 mM; $M_1:L_3$ = [Fe] = 0.42 mM, [Az] = 1.25 mM; $M_2:L_3$ = [Fe] = 0.80 mM, [Az] = 1.20 mM. $v$ = 10 mV $s^{-1}$, $E_{step}$ = 0.01 V. The arrow indicates the direction of the current.

**Table S2.** The tabulated reduction potential ($E_p$ vs Ag/AgCl 3 M NaCl) values and the corresponding peak currents ($i_p$) extracted from the cyclic voltammograms of different M:L ratio Fe(III) azotochelin solutions in NH4OAc buffer employed in native ESI-MS studies at pH 7.0.

| M:L | [Fe] / mM | [Az] / mM | $E_p$ / mV[a] | $-i_p$ / μA |
|---|---|---|---|---|
| 1:1 | 0.45 | 0.45 | –650 | 0.387 |
| 1:2 | 0.43 | 0.87 | –650 | 0.419 |
| 1:3 | 0.42 | 1.25 | –640 | 0.419 |
| 2:3 | 0.80 | 1.20 | –650 | 0.806 |

[a] $E_p$ vs Ag/AgCl 3 M NaCl; 5 mM $NH_4OAc$ buffer, 100 mM NaCl, pH 7.0; $v$ = 10 mV $s^{-1}$; $E_{step}$ = 0.01 V

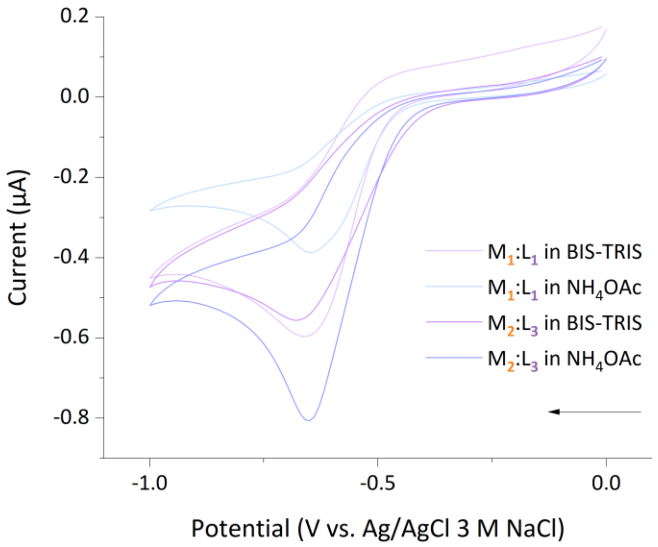


**Figure S5.** Cyclic voltammograms of a Fe(III) azotochelin solution in 5 mM buffer salt containing 100 mM NaCl at pH 7.0, where buffer is either BIS-TRIS (standard electrochemistry buffer) or $NH_4OAc$ (native ESI-MS buffer). Comparison of CVs between the two buffers at two metal (M) to ligand (L) ratios. $M_1$:$L_1$ = [Fe] = 0.45 mM, [Az] = 0.45 mM; $M_2$:$L_3$ = [Fe] = 0.80 mM, [Az] = 1.20 mM. $v$ = 10 mV $s^{-1}$, $E_{step}$ = 0.01 V. The arrow indicates the direction of the current.

## Native ESI-MS studies of Fe(III) azotochelin.

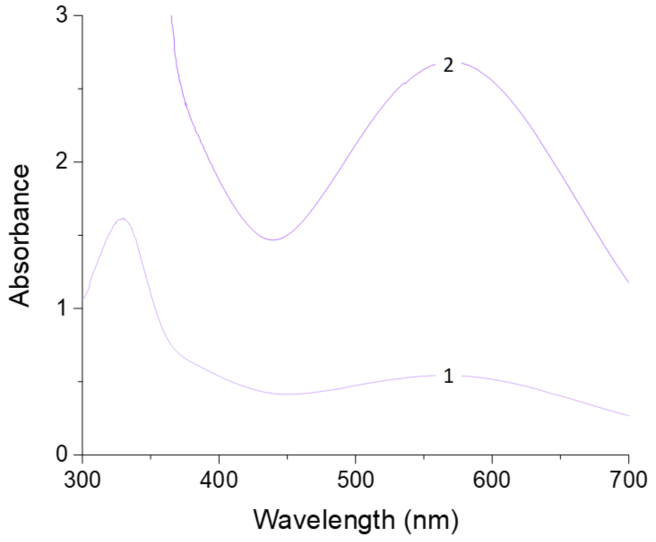


**Figure S6.** UV-vis absorption spectra of Fe(III) azotochelin samples submitted for native ESI-MS studies in 5 mM $NH_4OAc$ buffer, pH 7.0. (**1**) $M_1$:$L_1$, [Fe] = 0.45 mM, [Az] = 0.45 mM (**2**) $M_2$:$L_3$, [Fe] = 0.80 mM, [Az] = 1.20 mM.

**York - Chemistry - Mass Spectrometry Service Report**

*NGB-201013-C2-05 Fe k_akdk85704nb*

# A

**Analysis Information**

| | | | |
|---|---|---|---|
| Analysis Filename | akdk85704nb_P1-F-3_01_21707.d | Acquisition Date | 16/10/2020 07:07:18 |
| Method | ESI_low mass neg_2c1s.m | Instrument | compact |
| Submission Name | akdk85704nb | ESI | Negative |

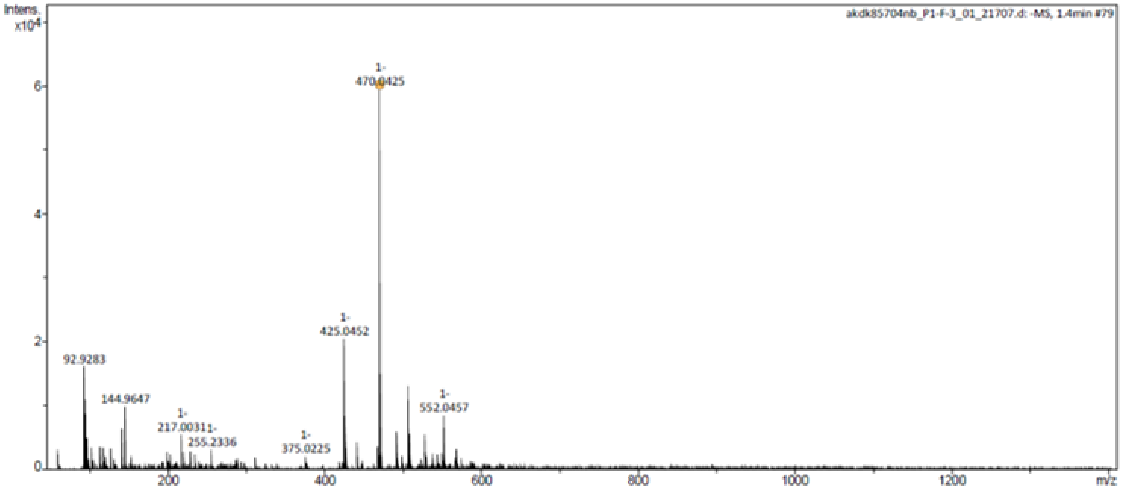


| Meas. m/z | # | Ion Formula | m/z | err [ppm] | err [mDa] | mSigma | Mean err [ppm] |
|---|---|---|---|---|---|---|---|
| 470.0425 | 1 | C20H18FeN2O8 | 470.0418 | -1.5 | -0.7 | 6.3 | 0.6 |

**York - Chemistry - Mass Spectrometry Service Report**

*NGB-201013-C2-05 Fe l_akdk85705nb*

# B

**Analysis Information**

| | | | |
|---|---|---|---|
| Analysis Filename | akdk85705nb_P1-F-4_01_21708.d | Acquisition Date | 16/10/2020 07:10:15 |
| Method | ESI_low mass neg_2c1s.m | Instrument | compact |
| Submission Name | akdk85705nb | ESI | Negative |

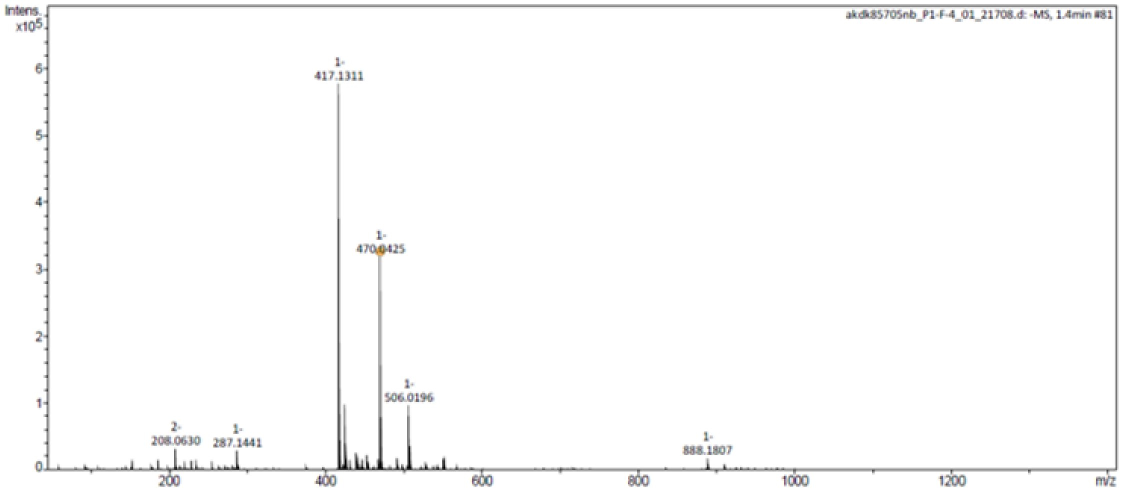


| Meas. m/z | # | Ion Formula | m/z | err [ppm] | err [mDa] | mSigma | Mean err [ppm] |
|---|---|---|---|---|---|---|---|
| 470.0425 | 1 | C20H18FeN2O8 | 470.0418 | -1.4 | -0.7 | 5.6 | -1.8 |

**Figure S7.** Native ESI(negative)-MS spectra of a Fe(III) azotochelin solution in 10 mM $NH_4OAc$ buffer, pH 7.0. (**A**) $M_1$:$L_1$, [Fe] = 0.45 mM, [Az] = 0.45 mM (**B**) $M_2$:$L_3$, [Fe] = 0.80 mM, [Az] = 1.20 mM.

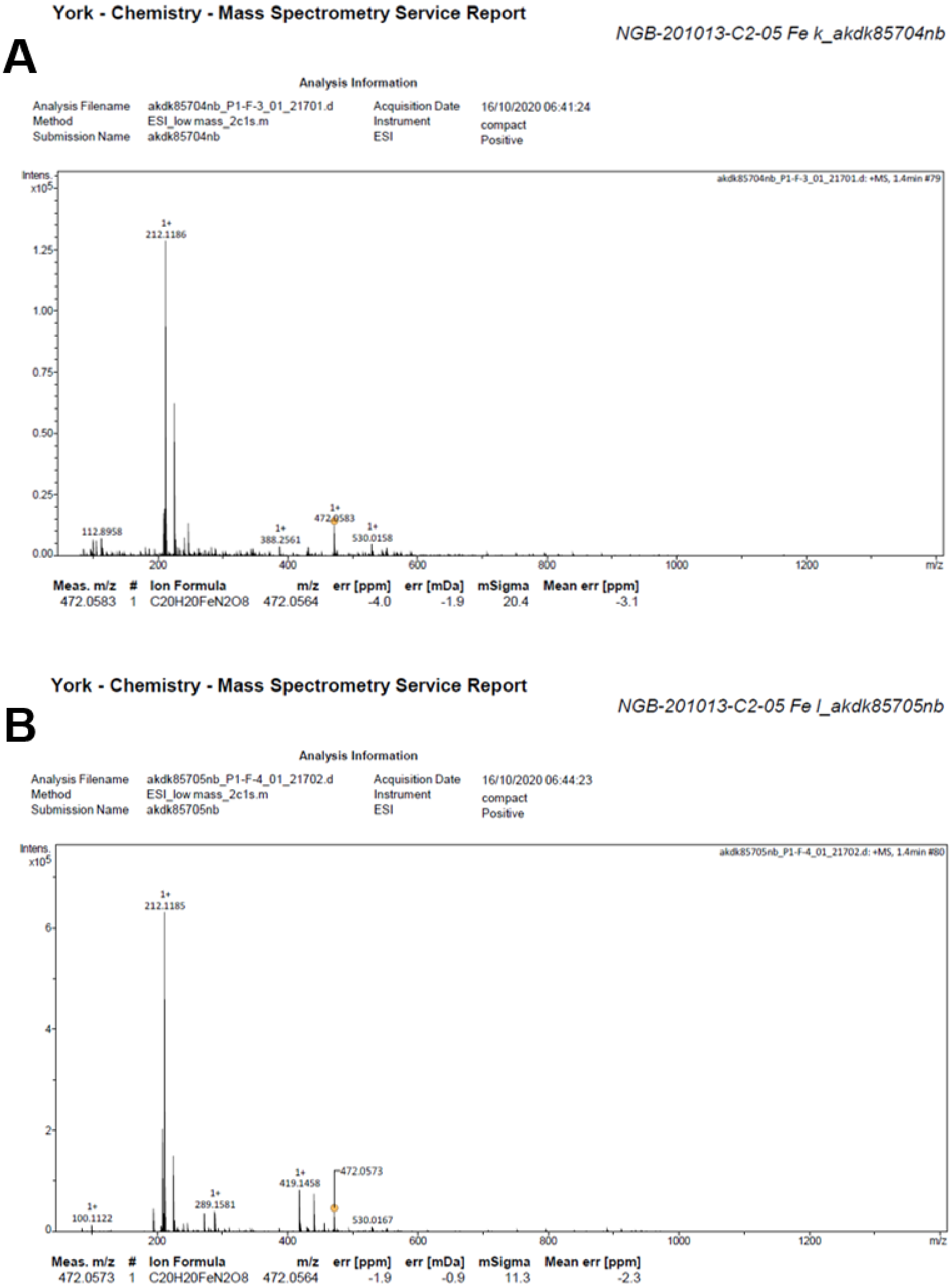


**Figure S8.** Native ESI(positive)-MS spectra of a Fe(III) azotochelin solution in 10 mM $NH_4OAc$ buffer, pH 7.0. (**A**) $M_1$:$L_1$, [Fe] = 0.45 mM, [Az] = 0.45 mM (**B**) $M_2$:$L_3$, [Fe] = 0.80 mM, [Az] = 1.20 mM.

**A**

**York - Chemistry - Mass Spectrometry Service Report**

*NGB-201013-C2-05 Fe g_akdk85700nb*

**Analysis Information**

| | | | |
|---|---|---|---|
| Analysis Filename | akdk85700nb_P1-E-8_01_21703.d | Acquisition Date | 16/10/2020 06:55:25 |
| Method | ESI_low mass neg_2c1s.m | Instrument | compact |
| Submission Name | akdk85700nb | ESI | Negative |

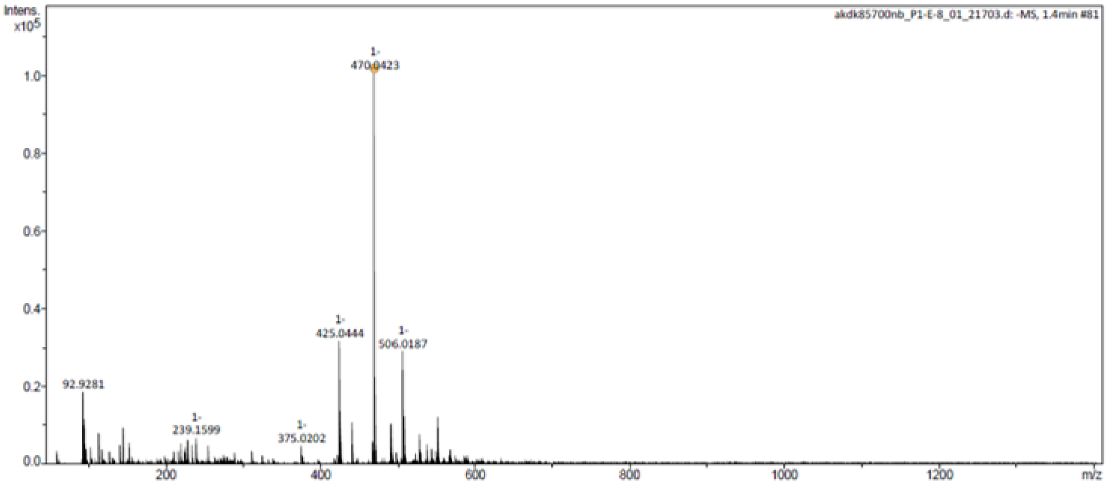


| Meas. m/z | # | Ion Formula | m/z | err [ppm] | err [mDa] | mSigma | Mean err [ppm] |
|---|---|---|---|---|---|---|---|
| 470.0423 | 1 | C20H18FeN2O8 | 470.0418 | -1.0 | -0.5 | 11.6 | -1.2 |

**York - Chemistry - Mass Spectrometry Service Report**

*NGB-201013-C2-05 Fe h_akdk85701nb*

**B**

**Analysis Information**

| | | | |
|---|---|---|---|
| Analysis Filename | akdk85701nb_P1-E-9_01_21704.d | Acquisition Date | 16/10/2020 06:58:24 |
| Method | ESI_low mass neg_2c1s.m | Instrument | compact |
| Submission Name | akdk85701nb | ESI | Negative |

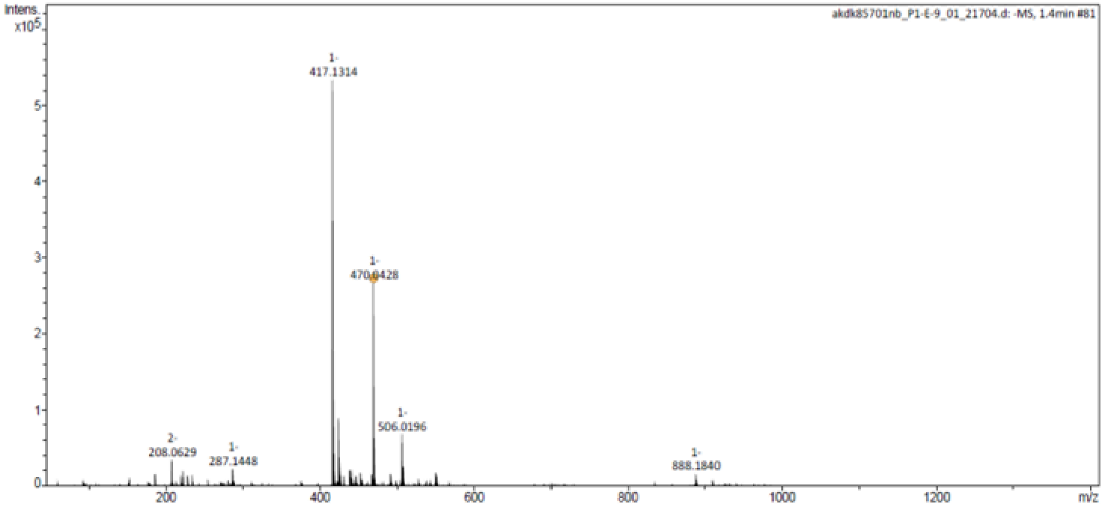


| Meas. m/z | # | Ion Formula | m/z | err [ppm] | err [mDa] | mSigma | Mean err [ppm] |
|---|---|---|---|---|---|---|---|
| 470.0428 | 1 | C20H18FeN2O8 | 470.0418 | -2.2 | -1.0 | 6.6 | -1.2 |

**York - Chemistry - Mass Spectrometry Service Report**

**C**

*NGB-201013-C2-05 Fe i_akdk85702nb*

**Analysis Information**

| | | | |
|---|---|---|---|
| Analysis Filename | akdk85702nb_P1-F-1_01_21705.d | Acquisition Date | 16/10/2020 07:01:22 |
| Method | ESI_low mass neg_2c1s.m | Instrument | compact |
| Submission Name | akdk85702nb | ESI | Negative |

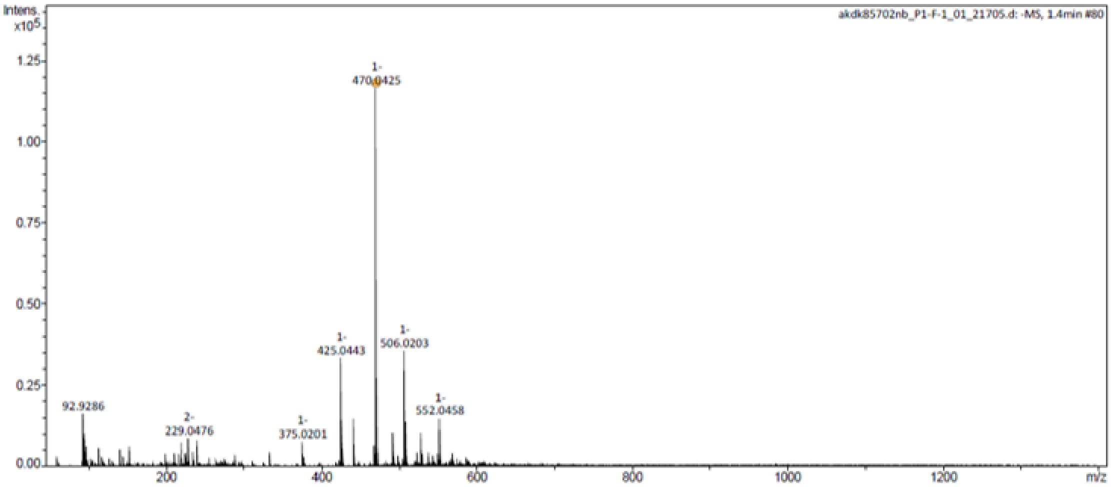


| Meas. m/z | # | Ion Formula | m/z | err [ppm] | err [mDa] | mSigma | Mean err [ppm] |
|---|---|---|---|---|---|---|---|
| 470.0425 | 1 | C20H18FeN2O8 | 470.0418 | -1.4 | -0.6 | 7.9 | -0.4 |

**York - Chemistry - Mass Spectrometry Service Report**

**D**

*NGB-201013-C2-05 Fe j_akdk85703nb*

**Analysis Information**

| | | | |
|---|---|---|---|
| Analysis Filename | akdk85703nb_P1-F-2_01_21706.d | Acquisition Date | 16/10/2020 07:04:20 |
| Method | ESI_low mass neg_2c1s.m | Instrument | compact |
| Submission Name | akdk85703nb | ESI | Negative |

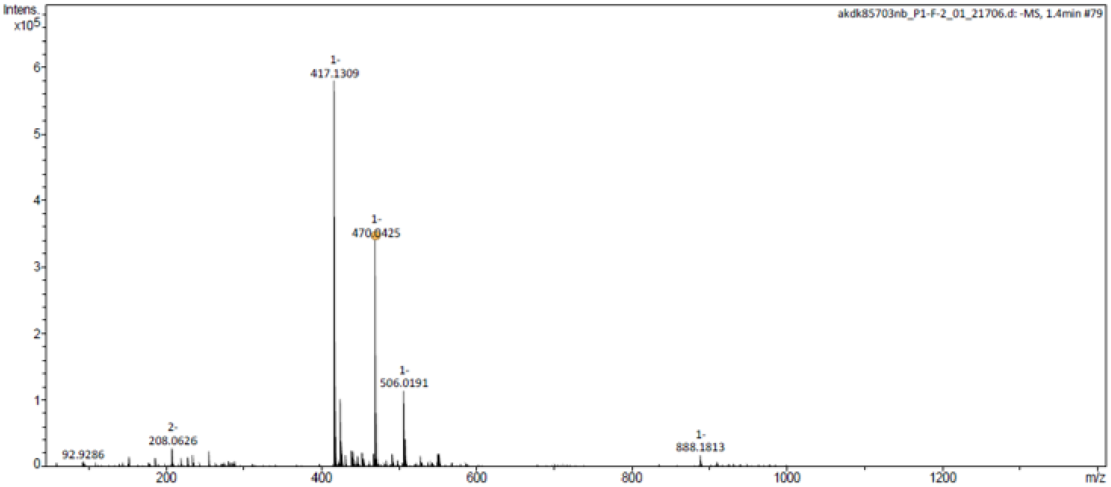


| Meas. m/z | # | Ion Formula | m/z | err [ppm] | err [mDa] | mSigma | Mean err [ppm] |
|---|---|---|---|---|---|---|---|
| 470.0425 | 1 | C20H18FeN2O8 | 470.0418 | -1.5 | -0.7 | 7.2 | -0.6 |

**Figure S9.** Native ESI(negative)-MS spectra of a Fe(III) azotochelin solution in 10 mM $NH_4OAc$ buffer. (**A**) pH 6.0, $M_1$:$L_1$, [Fe] = 0.45 mM, [Az] = 0.45 mM mM; (**B**) pH 6.0, $M_2$:$L_3$, [Fe] = 0.80 mM, [Az] = 1.20 mM (**C**) pH 6.5, $M_1$:$L_1$, [Fe] = 0.45 mM, [Az] = 0.45; (**D**) pH 6.5, $M_2$:$L_3$, [Fe] = 0.80 mM, [Az] = 1.20 mM.

## York - Chemistry - Mass Spectrometry Service Report

*NGB-201013-C2-05 Fe g_akdk85700nb*

**A**

**Analysis Information**

| | | | |
|---|---|---|---|
| Analysis Filename | akdk85700nb_P1-E-8_01_21697.d | Acquisition Date | 16/10/2020 06:29:28 |
| Method | ESI_low mass_2c1s.m | Instrument | compact |
| Submission Name | akdk85700nb | ESI | Positive |

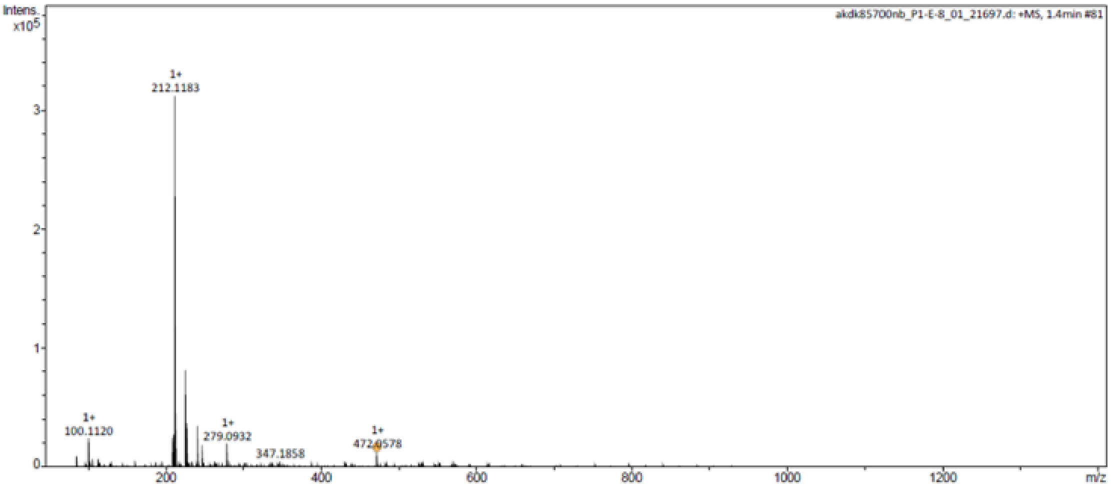


| Meas. m/z | # | Ion Formula | m/z | err [ppm] | err [mDa] | mSigma | Mean err [ppm] |
|---|---|---|---|---|---|---|---|
| 472.0578 | 1 | C20H20FeN2O8 | 472.0564 | -3.1 | -1.4 | 9.7 | -1.8 |

## York - Chemistry - Mass Spectrometry Service Report

*NGB-201013-C2-05 Fe h_akdk85701nb*

**B**

**Analysis Information**

| | | | |
|---|---|---|---|
| Analysis Filename | akdk85701nb_P1-E-9_01_21698.d | Acquisition Date | 16/10/2020 06:32:30 |
| Method | ESI_low mass_2c1s.m | Instrument | compact |
| Submission Name | akdk85701nb | ESI | Positive |

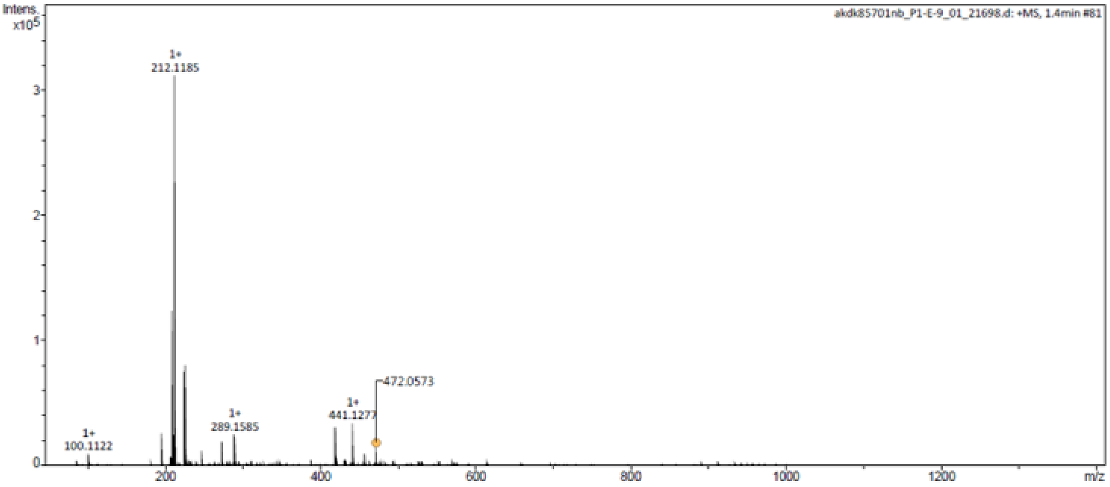


| Meas. m/z | # | Ion Formula | m/z | err [ppm] | err [mDa] | mSigma | Mean err [ppm] |
|---|---|---|---|---|---|---|---|
| 472.0573 | 1 | C20H20FeN2O8 | 472.0564 | -2.0 | -0.9 | 19.6 | -0.5 |

**C**

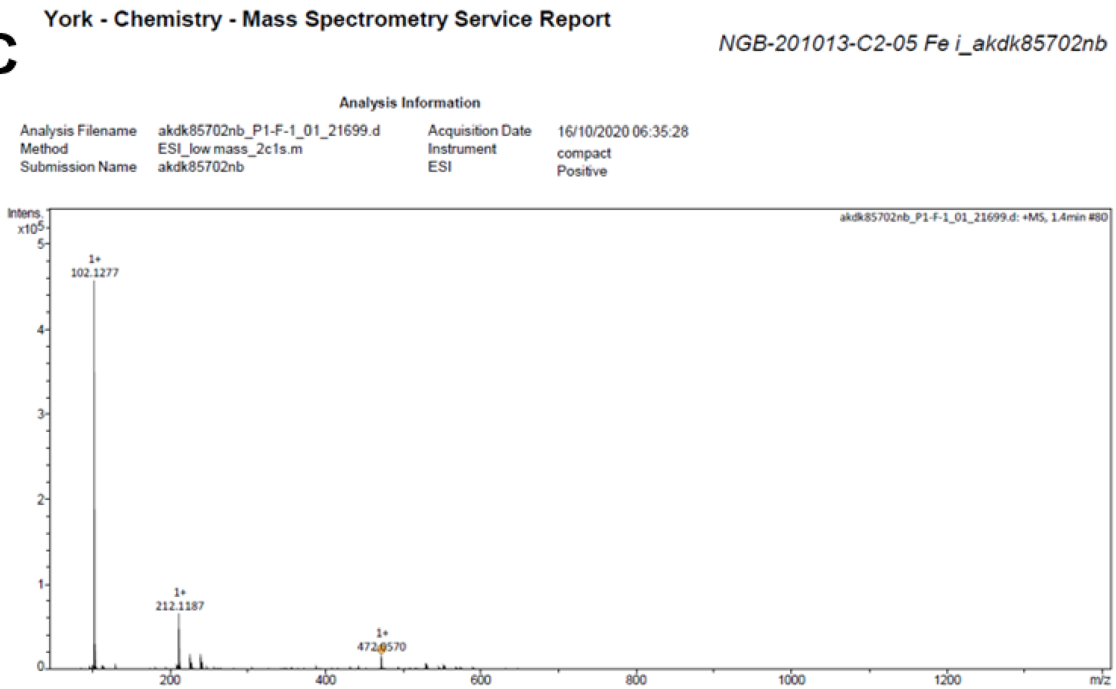


| Meas. m/z | # | Ion Formula | m/z | err [ppm] | err [mDa] | mSigma | Mean err [ppm] |
|---|---|---|---|---|---|---|---|
| 472.0570 | 1 | C20H20FeN2O8 | 472.0564 | -1.4 | -0.6 | 9.5 | -1.1 |

**D**

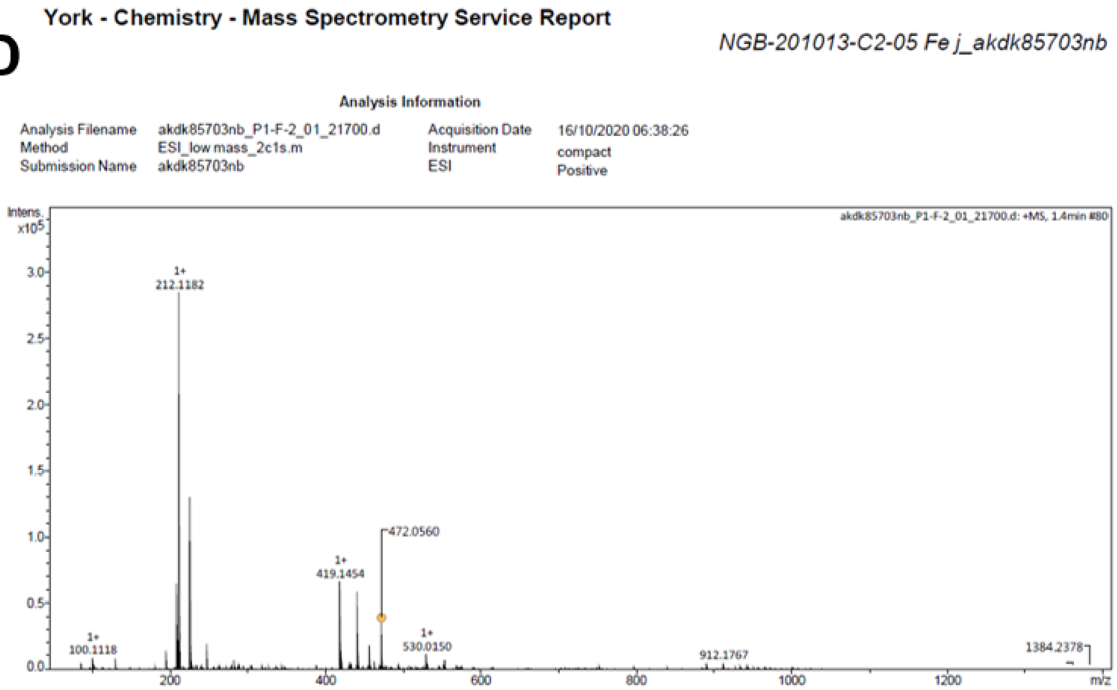


| Meas. m/z | # | Ion Formula | m/z | err [ppm] | err [mDa] | mSigma | Mean err [ppm] |
|---|---|---|---|---|---|---|---|
| 472.0560 | 1 | C20H20FeN2O8 | 472.0564 | 0.8 | 0.4 | 8.5 | -0.8 |

**Figure S10.** Native ESI(positive)-MS spectra of a Fe(III) azotochelin solution in 10 mM $NH_4OAc$ buffer. (**A**) pH 6.0, $M_1$:$L_1$, [Fe] = 0.45 mM, [Az] = 0.45 mM mM; (**B**) pH 6.0, $M_2$:$L_3$, [Fe] = 0.80 mM, [Az] = 1.20 mM (**C**) pH 6.5, $M_1$:$L_1$, [Fe] = 0.45 mM, [Az] = 0.45; (**D**) pH 6.5, $M_2$:$L_3$, [Fe] = 0.80 mM, [Az] = 1.20 mM.

**Buffer study data for Fe(III) azotochelin.**

Additional investigations into the buffer effect on the reduction potential of iron(III) azotochelin were inspired by previous reports which have noted this relationship for other siderophores. We hypothesise that the availability of the binding sites within the first coordination sphere of the coordinatively-unsaturated azotochelin complex enhances the buffer effect. We have selected buffers for this study based on their buffering abilities at pH 7.0 and the presence of either soft or hard donor atoms that could be capable of coordinating to the metal centre. Thus, all the buffers included in the investigation possess at least one nitrogen atom incorporated into their structure, albeit of varying pKa values, while some additionally include sulfonic acid groups, which under the experimental conditions employed, are deprotonated.[1] Below, we provide a more in-depth discussion about the results presented in Table 1.

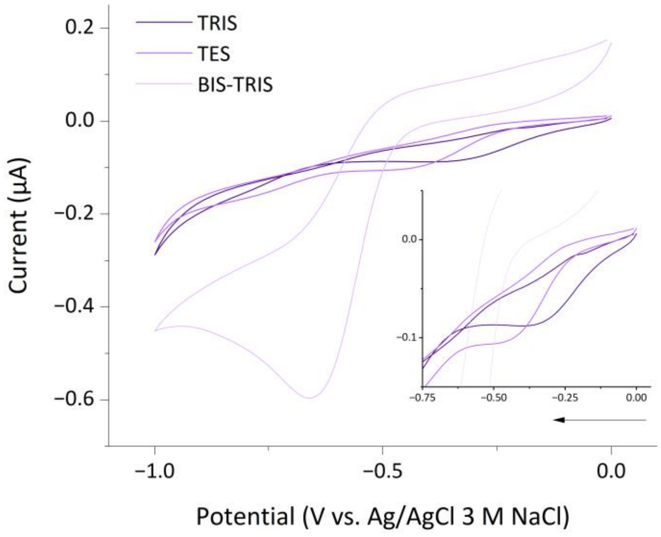


**Figure S11.** Cyclic voltammograms of iron(III) azotochelin ($M_1$:$L_1$) in different pH 7.0 buffer solutions (5 mM); all contain 100 mM NaCl as supporting electrolyte. Analyte concentrations of [Fe] = 0.45 mM; [Az] = 0.45 mM; $v$ = 10 mV $s^{-1}$, $E_{step}$ = 0.01 V. The arrow indicates the direction of the current. The inset provides enlargement of the reduction peak waves for the CVs in TRIS and TES buffers.

For the primary amine buffers, a larger $E_p$ shift observed in the TRIS buffer reflects the higher p$K_a$ value of its primary amine which results in its higher affinity towards the highly Lewis acidic iron(III) in comparison to the secondary amine in the TES buffer. Moreover,

the presence of sulfonic acid group in the TES buffer can limit the buffer's coordination ability to the metal centre due to increased steric bulk and electrostatic repulsion between the deprotonated oxygen of the sulfonic acid and the negatively-charged iron(III) azotochelin complex, further supporting the observed smaller $E_p$ shift as opposed to the TRIS buffer. A noticeable drop in Faradaic current is observed for the iron(III) azotochelin complex in the TES buffer, with a further decrease in TRIS, as compared to the BIS-TRIS buffer. We cannot determine whether these changes can be attributed to either steric or electrostatic effects as it is not possible to assign the exact charge of the complex under different buffer conditions without further mass spectrometric investigations, which are not possible due to the necessity to employ a volatile buffer, of which neither TRIS nor TES is.

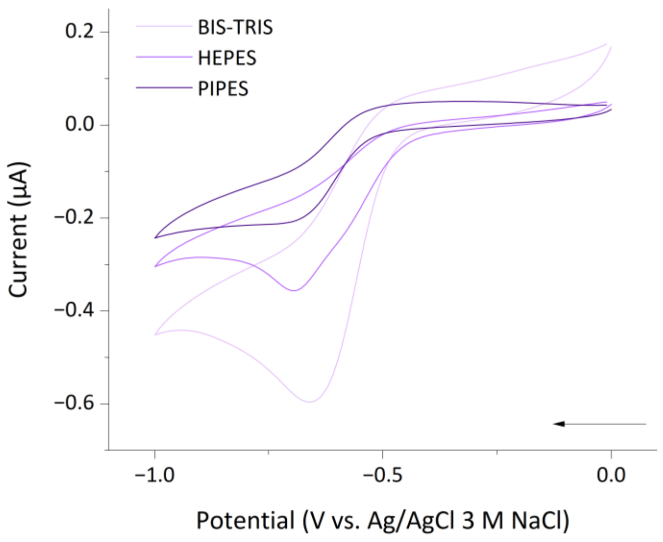


**Figure S12.** Cyclic voltammograms of iron(III) azotochelin ($M_1$:$L_1$) in different pH 7.0 buffer solutions (5 mM); all contain 100 mM NaCl as supporting electrolyte. Analyte concentrations of [Fe] = 0.45 mM; [Az] = 0.45 mM; $v$ = 10 mV $s^{-1}$, $E_{step}$ = 0.01 V. The arrow indicates the direction of the current.

We observed an opposite trend for HEPES and PIPES buffers (Figure S12), piperazine-based buffers containing tertiary amines, with a negative shift in the reduction peak potential of -40 mV and -50 mV versus $E_p$ in BIS-TRIS buffer, respectively. Analogous to BIS-TRIS, both HEPES and PIPES have been described in the literature to exhibit minimal or no metal-coordinating abilities, presumably due to the steric bulk around the donor atom and on average lower Lewis basicity of their amines as indicated by their p$K_a$

values. However, contrary to BIS-TRIS, HEPES and PIPES buffers comprise one and two sulfonic acid groups, respectively, which have the capacity for coordination to the metal cation through the deprotonated oxygen atoms, albeit the electronic repulsion between the negatively-charged buffer and redox-active species has to also be taken into consideration. The coordination of oxygen, a hard donor atom would justify the negative shift in the reduction peak potential of iron(III) azotochelin, as the interaction should stabilise the iron(III) complex subsequently making it more difficult to reduce the metal.

## CVs of Fe(III) tris(catecholate) complexes.

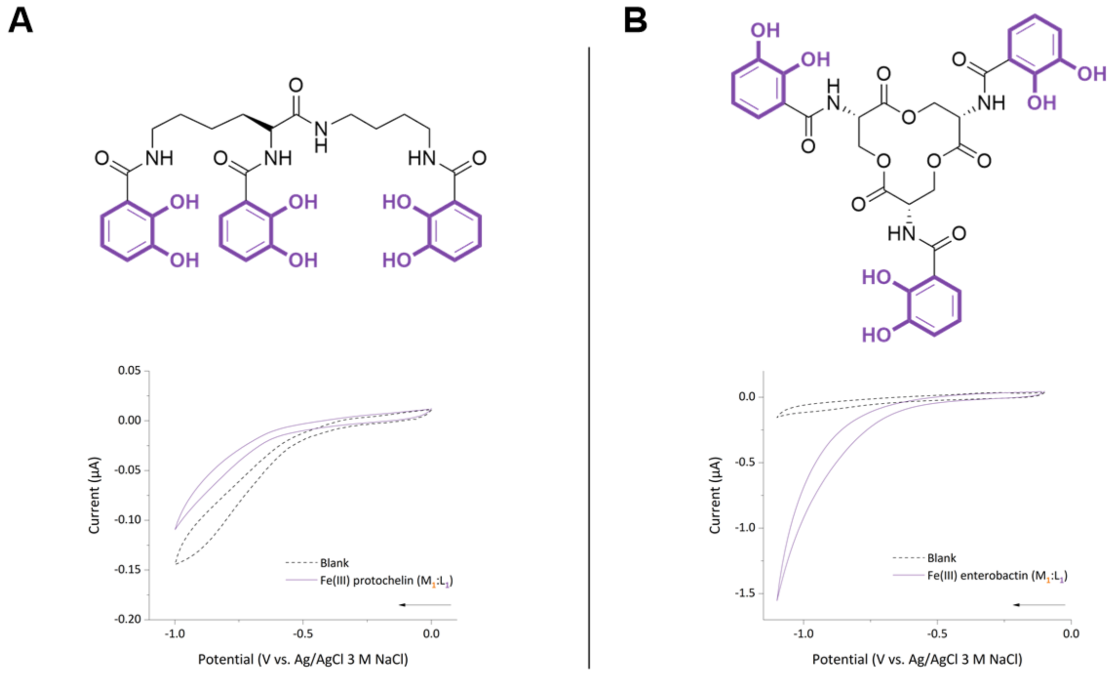


**Figure S13.** Chemical structures of the tris(catecholate) siderophores and cyclic voltammograms of solutions containing their iron(III) complexes in 5 mM BIS-TRIS buffer containing 100 mM NaCl. $E_{step}$ = 0.01 V. The arrow indicates the direction of the current. (**A**) Protochelin: $M_1$:$L_1$ = [Fe] = 0.42 mM, [Prot] = 0.42 mM; pH 7.5; $v$ = 10 mV $s^{-1}$ (**B**) Enterobactin: $M_1$:$L_1$ = [Fe] = 0.42 mM, [Ent] = 0.42 mM; pH 7.0; $v$ = 100 mV $s^{-1}$. The arrow indicates the direction of the current.

**BDD working electrode characterisation.**

The characterisation of the BDD working electrode was performed by studying the electrochemical behaviour of the potassium salt of ferricyanide, $K_3[Fe(CN)_6]$, and comparing it to the data obtained at a glassy carbon (GC) electrode (Table S3). From the larger peak-to-peak separation (ΔE) value and a shift in peak midpoint potentials ($E_{1/2}$) at different scan rates, it is clear that the BDD electrode exhibits different thermodynamics and kinetics of electron transfer between the electrode and the redox species. Moreover, we studied the effect of buffer on the electrochemical behaviour of ferricyanide at the BDD electrode (Table S4). We found it to have a further effect on the electron transfer as indicated by a shift in both the $E_p$ and ΔE values, highlighting the sensitivity of the BDD electrode to solution composition.

**Table S3.** The electrochemical data extracted from cyclic voltammograms of a $K_3[Fe(CN)_6]$ solution at two different scan rates (*v*), for comparison of two different working electrodes, a glassy carbon (GC) and boron-doped diamond (BDD).

| Working electrode | $v$ / mV s$^{-1}$ | $E_{1/2}$ / V$^a$ | ΔE / mV | $i_a/i_c$ |
|---|---|---|---|---|
| GC | 100 | 0.235 | 90 | 0.82 |
| | 50 | 0.235 | 90 | 0.81 |
| BDD | 100 | 0.230 | 120 | 0.78 |
| | 50 | 0.235 | 110 | 0.73 |

[a] $E_p$ vs Ag/AgCl 3 M NaCl; 100 mM phosphate buffer, 233 mM NaCl, pH 7.0; $E_{step}$ = 0.01 V.

**Table S4.** The electrochemical data extracted from cyclic voltammograms of a $K_3[Fe(CN)_6]$ solution at BDD working electrode in two different buffers to evaluate the effect of solution composition on the electrochemical behaviour of ferricyanide at the BDD electrode.

| Buffer | $E_{1/2}$ / V$^a$ | ΔE / mV | $i_a/i_c$ |
|---|---|---|---|
| Phosphate[b] | 0.235 | 110 | 0.73 |
| BIS-TRIS[c] | 0.205[d] | 90 | 1.22 |

[a] $E_p$ vs Ag/AgCl 3 M NaCl; $v$ = 50 mV s$^{-1}$; $E_{step}$ = 0.01 V.

[b] 100 mM phosphate buffer, 233 mM NaCl, pH 7.0; $[K_3[Fe(CN)_6]^{3-}]$ = 10 mM.

[c] 5 mM BIS-TRIS buffer, 100 mM NaCl, pH 7.0; $[K_3[Fe(CN)_6]^{3-}]$ = 0.48 mM.

[d] Comparable to the $E_{1/2}$ literature value of 0.206 V vs Ag/AgCl 3M KCl measured at a BDD working electrode in 100 mM sodium acetate buffer, pH 7.3.[2]

## Reference electrode conversion.

The CV data for ferricyanide at the GC electrode has also been used to determine the conversion factor (c.f.) between the Ag/AgCl 3 M NaCl reference electrode and the normal hydrogen electrode (NHE). The $E_{1/2}$ value (calculated as the average of the cathodic ($E_{pc}$) and anodic ($E_{pa}$) peak positions) for the ferri/ferrocyanide redox couple was compared to the literature to produce a c.f. of 0.190 V as described in Equation S1 with a literature value of 0.425 V vs NHE and an experimental value of 0.235 V vs. Ag/AgCl 3 M NaCl.[3]

Literature potential (V vs. NHE) = Experimental potential (V vs Ag/AgCl) + **c.f. (V)**

**Equation S1.** The potential conversion between two different reference electrodes as determined experimentally through cyclic voltammetry of 10 mM $K_3[Fe(CN)_6]$ in 100 mM phosphate buffer ($I$ = 0.464 M, NaCl) (pH 7.0).

## References.


(1) Good, N. E.; Winget, G. D.; Winter, W.; Connolly, T. N.; Izawa, S.; Singh, R. M. M. Hydrogen ion buffers for biological research. *Biochemistry* **1966**, *5*, 467–477.

(2) Weber, G.; von Wirén, N.; Hayen, H. Investigation of ascorbate-mediated iron release from ferric phytosiderophores in the presence of nicotianamine. *BioMetals* **2008**, *21*, 503–513.

(3) O'Reilly, J. E. Oxidation-reduction potential of the ferro-ferricyanide system in buffer solutions. *Biochim. Biophys. Acta, Bioenerg.* **1973**, *292*, 509–515.
